# Hierarchical, Interactive, and Dynamic Predictive Capacity of Current Biological, Psychological, Social, and Environmental Measurements in Depression, Anxiety, ADHD, and Social Quality across the Lifespan

**DOI:** 10.21203/rs.3.rs-7060126/v1

**Published:** 2025-07-30

**Authors:** Clark Roberts, Raphael Yamamoto, Zhafira Fawnia, Percy Mistry, Issac N Treves, Alexandra Decker, Samantha N. Sallie, Chris Chatham, Awais Aftab, Kim Lee, Madelynn Park, Vinod Menon, John Gabrieli

**Affiliations:** (1) McGovern Institute for Brain Research, Massachusetts Institute of Technology, Cambridge, MA, USA; (2) Department of Computer and Information Science, University of Pennsylvania; (3) Department of Computer Science & Neuroscience, Haverford College; (4) Department of Computer Science, Brown University Providence, RI, USA; (5) Department of Psychiatry & Behavioral Sciences, Stanford University School of Medicine, Stanford, CA; (6) Department of Psychiatry, Columbia University, New York City, NY, United States; (7) Department of Psychiatry, University of Cambridge, Cambridge, UK; (8) Medical Biomarker Research, Shionogi, Inc, Florham Park NJ, USA; (9) Department of Psychiatry, Case Western Reserve University, Cleveland, Ohio, USA

**Keywords:** Depression, Anxiety, ADHD, Social, Development, Transdiagnostic, Prediction, Cognition

## Abstract

An extensive and perplexing plurality of psychometric assesments, experimental-tasks, and biophysical measurement modalities have evolved alongside increasingly biopsychosocial models of behavior and psychopathology. Yet, despite alarming recent increasing rates of mental health problems, cumulative progress regarding the validity and *comparative* utility of wide-ranging measures to predict, isolate, or explain hallmark features and interacting systems in depression, anxiety, and ADHD remains concerningly enigmatic. Utilizing adolescent (Age 9–14) and parent data when available (combined N=~23,760) across 5 years from the ABCD dataset - we parcellated over a thousand unique biopsychosocial measures from all time points into 30 theoretically relevant, commonly-used, or potentially-informative domains. We then assessed their hierarchical and interactive predictive power using a novel integrative ensemble stacking of 4 state-of-the-art ML models. Further, addressing prior tradeoffs between high-dimensional prediction and interpretability, several synergistic methods were applied to provide uncertainty estimation, central features, and possible causal developmental dynamics. Results reveal notable overlapping transdiagnostic features as well as pronounced hierarchical differences between measurement-domains in predicting psychiatric, social, and cognitive outcomes. Myriad subjective-report metrics accounted for 27–76 fold more variation in ADHD, depression, and anxiety than an extensive available range of biophysical and experimental-task measurements. Still, many domain-specific features were highly predictive of several clinically meaningful operationalizations of depression and psychopathology. Illustrated are robust, consistent, and often bidirectional interactions between specific trait-like characteristics and dynamic features of coextensive psychopathology, somatic, social, and family relationships - accounting for substantial variation in ADHD, anxiety, and depression (r2=.49–.56). Psychiatric and social-quality targets in adults using available parent data exhibited similar hierarchical predictive patterns but also reveal important developmental differences. Collectively, these findings underscore the comparative predictive utility of wide-ranging current biopsychosocial measurements across several cardinal explanatory targets in psychiatry, and illuminate the structure and dynamics of the most prevalent mental-health challenges across the lifespan in novel detail, with implications for prediction, explanation, and control.

## Introduction

With considerable and still unclear cross-cultural variation (GBD, 2022), some epidemiological studies estimate the lifetime risk of Major Depressive Disorder (MDD) around 5–28% (Kruijshaar et al., 2005; Kessler et al., 2012; Tam et al., 2020; Bains & Abdijadid, 2023), and approximately 30% for anxiety disorders (Kessler et al., 2012; Bandelow et al., 2015;), with their prevalence markedly exceeding other current diagnostic categories in psychiatry (WHO, 2023). Attention-Deficit/Hyperactivity Disorder (ADHD) is also comparatively common, affecting approximately 5–11% of people (Danielson et al, 2024; NIMH, 2023). With notable variation, median approximations for formal diagnosis are around 30, 17, and 12 years of age for depression, anxiety and ADHD, respectively (Solmi et al., 2022). However, dimensionally, their hallmark features (e.g., chronic low-mood/anhedonia, worry/fear, attention dysregulation) can arise much earlier in development within subclinical ranges and are strongly predictive of later formal diagnosis (Wakefield et al, 2017; Aftab & Druss, 2023; McGorry et al, 2024). Alarmingly, considerable recent evidence indicates rates of all such categorical diagnosis appear to be increasing in children, adolescents, and young adults (Goodwin et al, 2022; Kreski et al, 2022; Keyes et al, 2024), propelling researchers to grapple with multifaceted questions about etiology, measurement, and societal influences.

Substantial covariation between their hallmark features, including personality dimensions (Hopwood et al, 2022; Waszczuk et al, 2022; Soodla et al, 2025), as well as frequent (often antecedent) co-morbidity rates (Kessler et al, 2012), indicate perplexing and still unclear diagnostic boundaries and interactions - appearing to often occur in a nexus of self-reinforcing symptoms and behaviors (Borsboom et al, 2019; Uhlhaas, et al, 2023; Forbes et al, 2024; Njardvick et al, 2025). Uncertainty and debates persist about when and whether some diagnostic hallmark features should be viewed as dimensions or categories (Gillan & Seow, 2020), how autopoietic pathosuggestive dynamics are neurodevelopmentally engendered and with what degree of etiological variability, and when or even if they can be effectively predicted or explainable through more reductive and objective biophysical measures (Ghaemi, 2009; Jefferson, 2022; Liu & Lau, 2022; Aftab, 2024; Barrett et al, 2024; Blithikioti et al, 2025).

Depression particularly has been at the center of such controversies with a now diverse range of public conjectures (Bridger et al, 2025) and often politically-charged beliefs about its causes. Variable researcher perspectives (Sulik et al, 2025) and past results regarding attributable causal or even associative relationships have similarly engendered some contention alongside repodroducibility challenges. Central challenges and uncertainty has persisted regarding the ontology and variability in diagnostic classification, relative influence of psychosocial and environmental factors (Hagan & Syme, 2024), concerns regarding the validity and extensive diversity of experimental paradigms (Firestone & Scholl, 2015; Eisenberg et al, 2019; Serra-Garcia & Gneezy, 2021; Kervelis et al, 2022; Corneille & Gawronski, 2024) to capture latent facets of explanatory targets in psychiatry (Michel, 2024), intervention efficacy and case-control experimental task differences sometimes appearing as non-specific transdiagnostic features (Abramovitch et al, 2021; Haeffel et, 2021), inflated extrapolations from p-values despite minimal effect sizes in some underpowered studies, and general biological and causal complexity (Ross, 2022; Pessoa, 2025; Krakauer, 2024; Potter & Mitchell, 2025). Some folk psychology constructs remain difficult to ground scientifically (Hommel et al, 2019; LeDoux, 2021) and there is an ongoing risk of conflating of statistical models with theoretical models, particularly in psychiatry (Fried, 2020). Despite a proliferation of biopsychosocial measures and corresponding theories, cumulative progress to predict real-world outcomes or provide a unified multidisciplinary empirical grounding of several central constructs (Eisenberg et al, 2019) remains convoluted.

In an attempt to address ongoing multifaceted scientific uncertainty and evaluate the relative predictive capacity, utility, and interactions of an extensive range of current measures, we utilized the scope of the ABCD dataset, including both adolescent and available adult/parent data. Proportional to sample size, the range of biological, psychological, social, and environmental measurements collected within ABCD is unparalleled. Our primary goals within were to 1.) Systematically parcellate the breadth of the dataset at each time point into ontologically relevant or pragmatically informative categorical domains 2.) comparatively and hierarchically assess predictive capacities *within* categories 3.) determine most predictive features and robust interactions *across* categories 4.) apply complementary analysis to assess central, dynamic, and possibly causal features in developmental psychopathology. Given notorious methodological tradeoffs, towards this aim, we developed an analytic pipeline to balance exploratory high-dimensional prediction with more theory-driven models directed at coarse-grained interpretability. For further triangulation, validation, and comparison, we evaluated predictive models across several operationalizations of low-mood and depression (dimensional, chronic-trait, dynamic, severe, syndrome), alongside other paradigmatic features of the internalizing/externalizing spectra - which despite extensive covariation are frequently examined in isolation.

## Results

The ADHD/bad-grades/depression subtype (R^2^ = 0.557, Adjusted R^2^ = 0.482) shared social problems as a defining predictive feature but was more uniquely characterized by oppositional defiance, and potential externalizing pathways to depression. In contrast, while the high-achieving perfectionism/good-grades/depression/anxiety subtype (R^2^ = 0.471, Adjusted R^2^ = 0.380) similarly shared stubbornness as the dominant predictor, top-features were more specific to school-specific fears and anxiety.

Notably, the delta measure of social problems emerged as a strong predictor across multiple subtypes, aligning with our Bayesian mixed model findings (Fig 7) and further illustrating how changes in social relationship dynamics significantly influence mood fluctuations across different manifestations of internalizing symptoms.

B. Right side includes ensemble feature importance rankings across all categories for low-mood/depression in high/low ALE history and male/female stratifications reveal distinct predictive patterns. When stratified by adverse life event (ALE) history, individuals with low ALE exposure (top left, N=1,226, R^2^ = 0.389) showed recent social problems and temper tantrums as top predictors of depression. In contrast, those with high ALE history (top right, N=1,628, R^2^ = 0.558) displayed anxiety-related features as primary predictors, including worries, paranoia, and embarrassment sensitivity.

Gender stratification (bottom panels) showed both several similarities with some important distinctions. In females (N=3,464, R^2^ = 0.464), fear of school emerged as the strongest predictor of low-mood/depression, followed by general nervousness and stubbornness. This pattern suggests overlap with the high-achieving perfectionism subtype seen in our other analyses. Males (N=4,026, R^2^ = 0.455) showed a relatively similar profile.

Across all stratifications, several overlapping specific trait-like characteristics and co-extensive or changing anxiety and social relationship quality again consistently appeared as top predictors, largely indicating their central role in mood outcomes regardless of gender, life event history, or subtype. Across-category feature importance metrics for parent suicidal ideations are also included in supplementary materials S.3.4.)

Results indicate a striking discrepancy in predictive capacity: parent-to-child subjective ratings consistently demonstrated the highest predictive accuracy for psychopathological outcomes (ADHD, anxiety, depression), whereas entirely objective measures exhibited minimal predictive accuracy. Relative to other psychopathological outcomes, ADHD exhibited modestly higher predictive associations with measures at the more objective end of the continuum. Educational performance outcomes, however, showed comparable predictive strength from objective measures and parent-self-report. Surprisingly, child-self ratings on social relationships showed limited predictive associations with parent-rated mood outcomes, raising considerations about scale quality, inter-rater consistency, or genuine divergence between child-reported experiences and observed psychopathology. Categories with insufficient or overly circular variables were excluded to maintain predictive validity. Notably, child self-ratings of social relationship quality exhibited minimal associations with parent-rated child mood outcomes. Comprehensive details on the variable groupings are provided in supplementary materials S5.1. Full detailed ML evaluation and feature importance metrics for this categorization scheme are also provided in supplementary materials S.5.1.)

Parent self-rated version of CBCL notably has some different phrasing and measurement targets. However, similar to several hierarchical patterns in children, results indicate consistently strong predictive capacity of parent self-rated cognitive/attention issues, anxiety, personality traits, impulsivity, and recent changes in psychopathology (delta psychopathology). Child-rated psychopathology, particularly externalizing, mood, and anxiety, also showed notable predictions in parent mood, highlighting meaningful parent-child psychological interactions, further illustrated below (Fig 6, 8). Similar to child mood outcomes, objective or demographic measures (genetic ancestry, SES, residential characteristics, religion) provided minimal predictive power. Despite a wide set of available variables, parent drug use surprisingly showed minimal associations with parent mood. See supplement S1.1 or S3.1 for full within-category feature-sets used and full details feature importance rankings.)

In **children**, delta variables (panel A) show recent changes in social problems were most strongly associated with mood fluctuations, followed by somatic complaints, ADHD symptoms, adverse life events, and family conflict. For non-delta psychopathological syndrome measures (panel C), social problems, somatic complaints, and aggressive behaviors showed the strongest associations, while rule-breaking behaviors had minimal relationship with mood outcomes. Among non-psychopathological variables (panel E), parental divorce and family conflict emerged as the most reliable correlates of child mood, while frequently discussed variables such as social media use, fitbit-measured activity, and cognitive task parameters (TB Flanker, NB Correct) demonstrated minimal associations. Sex and religious importance showed negligible relationships with mood outcomes.

In **parents**, delta-change variables (panel B) reflecting feelings of being unloved, somatic complaints, and worries about the future showed the strongest associations with mood fluctuations, followed by friendship troubles and poor work performance. Other psychopathological/social measures (panel D) revealed somatic problems, feelings of being unloved, and worries about the future as key correlates, with child internalizing disorders also showing substantial relationships with parent mood. Among broader measures (panel F), frequent family conflict strongly emerged as the strongest association. Parent income negatively correlated with low mood, highlighting economic factors’ greater relevance for adults compared to children.

Overall, Bayesian mixed-model results align closely with prior machine-learning findings, providing enhanced uncertainty quantification, robustness to missing data, and the advantage of repeated-measures analysis. Results again underscore social relationships, somatic issues, and co-occurring psychopathological features as central and often dynamic correlates of mood. See BMM supplement S6.2 for ADHD outcomes contrast.)

### Household income relationship with parental internalizing and externalizing psychopathology

Two one-way ANCOVA models controlling for parental age, marital status, education level, sex, and the interaction between household income and parental sex showed significant main effects of household income on both parental internalizing (F(6, 8559)= 7.53, p< .001) and externalizing (F(6, 8559)= 2.11, p= .05) psychopathology. Post hoc tests revealed inverse relationships between household income and psychopathology types; those with the lowest household incomes demonstrated the highest levels of psychopathology, with a notable drop in both types of psychopathology observed in those within the highest recorded yearly income (i.e., 200k+) group (see Supplemental materials S6.3). Importantly, the relationship of household income was particularly prominent for the internalizing (η-p2= .005) compared to the externalizing (η-p2= .001) subtype in parents.

### Secondary outcomes on parental internalizing and externalizing psychopathology

We further showed significant main effects of parental age (F(1, 8559)= 7.28, p= .007) and marital status (F(1, 8559)= 5.14, p= .02) on parental externalizing psychopathology, with younger and unmarried parents more likely to exhibit problematic externalizing behaviours. Female parents at any household income level were more inclined to internalizing psychopathologies (F(1, 8559)= 5.63, p= .02) until surpassing the 200k income mark (whereby females and males demonstrated statistically equal levels of internalizing behaviour). We did not observe any significant interaction effects of household income and parental sex on either psychopathological subtype in parents using the whole economic cohort (p> .05). However, targeted ANCOVA analyses comparing parent sex differences in psychopathology specifically between the two highest household income group levels (i.e., 100–200k and 200k+) showed that female parents had less internalizing (F(1, 4077)=3.09, p= .05) and externalizing (F(1, 4077)= 5.39, p= .02) psychopathologies than male parents when their household income exceeded 200k annually compared to their 100–200k counterparts (see Supplemental materials S6.3).

### Household income relationship with child internalizing and externalizing psychopathology

Likewise, two one-way ANCOVA models controlling for parental variables: age, marital status, and education level; and child variable: sex- as well as the interaction between household income level and child sex- showed significant main effects of household income on both internalizing (F(6, 21280)= 20.76, p< .001) and externalizing (F(6, 21280)= 28.78, p< .001) psychopathology in children, with post hoc tests showing similar inverse directionality to their parents (see Supplemental materials S6.3). However, in contrast to their parents, household income showed a more profound inverse effect on child externalizing (η-p2= .008)- as opposed to internalizing (η-p2= .006)-behaviours.

### Recent negative income dynamics on parental internalizing and externalizing psychopathology

Using the same covariates, the effects of recent negative changes in income were contrasted by the effects of annual household income on parental psychopathology, with recent negative income dynamics associated more strongly with internalizing (F(6, 8561)= 296.96, p< .001, η-p2= .038) than externalizing (F(6, 8561)= 222.22, p< .001, η-p2= .025) in parents. These effects of recent negative dynamics in family income on parental psychopathology were particularly robust in medium (i.e., $35–50k and $50–75k) annual income groups.)

## Discussion:

Within a uniquely large and joint sample of both adolescents and adults – in part with the most extensive diversity of biological, psychological, social, and environmental measures currently acquired simultaneously and longitudinally – results here most pressingly reveal 1.) robust predictability can be afforded through specific measures, categorical domains, and interactions for the most common hallmark psychiatric features and several clinically relevant operationalizations and 2.) pronounced predictive variation between many pertinent *current* measurement domains intimated across the spectrum of biopsychosocial models of psychopathology. The following discussion first synthesizes key results, then overviews the most robust and consistent predictive relationships identified across analyses, and is followed by an examination of measurement-domains with concerningly limited predictive utility, collectively offering several implications for measurement, prediction, explanation, and likely control in developmental psychopathology. With some caveats mentioned below, overarching results here include:

1. **Model Performance and Prediction in Longitudinal Mood Outcomes:** Our novel ML ensemble stacking method, rigorously evaluated through nested cross-validation, yielded robust performance for all psychiatric targets (Fig 3,5), with mood and depression outcomes being strongly and consistently predicted across categories in parents (R^2^ = 0.443–0.467) and children (R^2^ = 0.502–0.605) throughout development. Across targets, substantial overlap *within* the most predictive categorical domains appears highly suggestive of risk/protective factor multifinality, including specific features *across* domains concurrently indicating concurrent equifinality. The resulting gestalt across a wide-range of demographic and stratification profiles (Fig 6) is that internalizing outcomes appear strongly empirically related to specific trait-like characteristics (personality, psychopathology, interpersonal, somatic-problems), often interacting with dynamics in other psychopathological dimensions, health/somatic issues, and social relationship quality. Notably, personality features—particularly proxies of cognitive rigidity, sensitivity to social feedback, and goal-directed efficiency—appear increasingly predictive of mood across development (Fig 4). Many of the consistent specific traits implicated appear theoretically related to cognitive facets which render ongoing narrow and ineffectual goal-directed behaviors (DeYoung & Krueger, 2018), in turn amplifying vulnerabilities to internalizing, particularly when coinciding with negative interpersonal feedback. Correspondingly, across *all* domains assessed, changes in social relationship quality and somatic issues (Fig 6,7) were most predictive of dimensional and clinically relevant shifts in mood and depression symptoms. Despite substantial overlap, features of social-quality showed consistently higher predictive power for mood than for anxiety outcomes (Fig 4), indicating an important theoretical difference in how social dynamics influence qualitatively different forms of internalizing.
2. **Alternative Target Contrasts:** Extensive domain-specific variation was observed across other psychiatric, social, and cognitive targets, with several notable similarities and important differences (Fig 3,4,5). Additional converging results illustrated consistent and robust empirical relationships between specific personality features and the frequently covarying psychopathology dimensions algorithmically captured within the P-factor (Kotov et al 2021; Hopwood et al, 2022) (Fig 3,3b,4). In particular, chronic attention-seeking behavioral styles in adolescents strongly predicted social problems, which in turn robustly related to internalizing. These findings suggest that while certain trait-characteristics are not present in widely used psychiatric diagnostic or classification schemes, they nonetheless may provide substantial prognostic mental-health information (Waszczuk et al, 2022). Such traits may reflect pre-existing vulnerabilities that, when coupled with aversive feedback, may generate deleterious feedback loops.
3. **Child-Parent Similarities/Differences:** For mood/depression outcomes, parent data indicated similar hierarchical predictive patterns to those observed in children across many specific features and broader categories (Fig 3,4,5), including self-reported personality traits, interpersonal problems, somatic symptoms, anxiety, attention difficulties, and broader features of cognition (Fig 5,6). Self-reported attention and cognitive issues in parents most strongly predicted co-extensive internalizing. Beyond depression, fluctuations in (and interconnections between) broader psychopathological features appeared to be largely influenced by similar ADHD-related features across children, parents, and their family-level interrelations (CLPN). A notable contradistinction is that objective family income showed disproportionately stronger effects on child externalizing and parent internalizing, wherein recent financial dynamics demonstrated even stronger relationships than objective income (F7ex).
4. **ALE:** ALE’s have shown variable but sometimes strong associations with depression in adults, particularly around the average age of onset for MDD (Kender et al, 1999; Keller et al, 2007; Solmi et al, 2022; Baldwin et al, 2023; Grummit et al, 2024). In the present study, ALE history in children demonstrated only modest relationships with depression outcomes (Fig 4,7), consistent with prior findings in the ABCD dataset (Brieant et al, 2023). Feature importance metrics indicated variation was largely driven by the degree to which participants reported being subjectively affected by event(s) (See supplementary materials S3.1). However, the early developmental stage of the cohort, the likelihood of uncaptured or unreported ALEs, and ambiguity regarding the magnitude of specific events make strong inferences challenging. However, aforementioned relationship quality and conflict, which can be regarded as a particular form of adversity, did show consistent and strong relationships with internalizing and changes therein.
5. **Underperforming Predictors/Categories:** With some exceptions, domains including current biological, environmental, technology-use, residential, ancestral/sociological, religious affiliation/practice, family drug-use, and experimental task measures showed limited capacity to predict psychiatric outcomes (particularly internalizing) in children (Fig 3,4), and parents - where data were available (Fig 5). Still, several measures within some categories had consistent (albeit small) correlations, modest developmental trends, and comparatively stronger relationships with alternative targets, specifically child grade and reading outcomes (Fig 3,4B). Notably, despite showing some predictive value for each other and for academic outcomes, the extensive array of experimental task parameters and multimodal neuroimaging features demonstrated minimal predictive capacity for any psychiatric outcomes—including ADHD (F3).
6. **General Objective/Self-Report Measure Discrepancy:** Despite rapid and promising technological advancements, our results demonstrate a striking predictive discrepancy between the spectrum of more or less ‘objective’ and ‘subjective’ measures across most psychiatric targets (Fig 4c). Parent-to-child rated measures (mostly personality/psychopathology/social) were highly predictive of CBCL Syndrome variables (Depression R^2^=0.591, Anxiety R^2^=0.533, ADHD R^2^=0.598). In contrast, a wide array of objective measures—comprising cognitive tasks, neuroimaging, ancestral gene PCS, and ‘physiological/activity’ fitbit data —yielded substantially lower predictive accuracy for the same outcomes (Depression R^2^=0.022, Anxiety R^2^=0.007, ADHD R^2^=0.040), reflecting a 27-fold difference for Depression, 76-fold for Anxiety, and 15-fold for ADHD. Interestingly, this discrepancy did not hold for academic outcomes: grade performance was predicted with comparable accuracy by objective measures (R^2^=0.213) and subjective parent ratings (R^2^=0.229).

### General Psychopathological Interactions

Low mood and anxiety, including proximal transistions between such states, are difficult to experimentally isolate scientifically given precipiating factors are temporally and idiographically complex. Many general psychopathological interactions observed in this study align with dynamic system and cybernetic models of psychopathology (Borsboom & Cramer, 2013; DeYoung & Krueger, 2018), which emphasize the role of internally weighted goal-directed pursuits and connections to social-environmental feedback. While some otherwise adaptive traits may, under certain mismatched or happenstance conditions, lead to negative outcomes (Nesse, 2019), our results suggest that internalizing and other mental health dimensions are often exacerbated (or even in large part shaped) by coextensive personality traits (Hopwood et al., 2022; Waszczuk et al., 2022; Soodla et al., 2025) and maladaptive behaviors that compromise efficacious goal pursuits and increase the delta between current and desired states.

The domain-specific category which most robustly accounted for variation in parent anxiety and depression contained features related to attentional and cognitive efficacy, particularly difficulties in planning. In the broader developmental nexus of psychopathology, illustrated in CLPNs containing other hallmark CBCL symptom clusters, ADHD showed the highest in- and out-strength across children, parents, and familial interactions (Fig 8), highlighting the centrality of attentional control systems in psychopathology, and suggesting their dysregulation as both cause and consequence of other mental health issues.

Clinical features of ADHD often developmentally precede depression (Hitchcock et al, 2022) and can generate negative outcomes in a wide range of educational, vocational, and social domains, which in turn contribute to negative affect (Swanson et al, 2024; Riglin et al, 2021; Solmi et al, 2021). Social problems were robustly predicted across categories (r2=.565), with ADHD and personality traits—particularly impulsivity and attention-seeking—emerging as key predictors in children (Fig 3b) and parents (Fig 5).

Some have recently speculated about the potential viable evolutionary utility of ADHD features in certain contexts, proposing adaptive benefits in flexibility, exploration, foraging, novelty-seeking, or creativity (Barack et al, 2022; Ivancovsky et al, 2024). However results here indicate, at least under the demands of contemporary contexts, features of ADHD are a central and exacerbating element in the nexus of many other mental health problems and cognitive efficacy across developmental stages. These results again underscore attentional dysregulation as a critical potential target for intervention (Roberts et al, 2023).

### Hierarchical Predictive Capacity Across Domains - Depression and Anxiety

Several specific features and broader categories demonstrated stable predictive validity for mental health challenges, particularly depression (Fig 3–6). These results reinforce that mood and anxiety are rarely orthogonal to other mental health dimensions, personality features, and dynamic psychosocial feedback (Fig 2,3,4,5,8). Despite considerable overlap, there were some theoretically important differences seen among in different operationalizations of mood—particularly between more stable trait-like patterns and recent clinical changes (Fig 3,5,7). Dynamic features, especially interpersonal and somatic changes, appeared influential in driving clinically relevant symptom transitions (Fig 6), whereas trait-like characteristics appeared strongly influential in amplifying dispositional vulnerabilities towards aversive state transitions (fig 3–6).

Notably, the proportion of variance in mood outcomes explained by personality or other trait-like characteristics increased consistently across developmental stages (F4). Some specific personality measures showed robust and consistent associations across various mood operationalizations and subtypes (Fig 4b, 6), including (1) stubbornness, (2) embarrassment and jealousy sensitivity, (3) attention-seeking, and (4) other paradigmatic ADHD-related features such as goal/task discontinuity. We speculate these features carry predictive utility for theoretically important reasons, including as proxies of maladaptive cognitive flexibility and reappraisal, interpersonal skills, heightened sensitivity to negative social feedback, and low tolerance to deviations or poor efficacy in goal-directed behavior - particularly within contemporary normative demands. Consistent with this, a recent large-sample study in adults found similarly nuanced personality features to be hierarchically and strongly associated with similar psychopathological dimensions (Soodla et al, 2025).

Perhaps unsurprisingly, anxiety symptoms—especially changes therein—were highly predictive of mood disturbances, consistent with well-documented comorbidity rates (Kessler et al, 2012). Anxiety and mood disorders have substantial comorbidity rates for likely several straightforward reasons. These overlaps likely reflect shared dispositional traits which appear at least partially stable over time—particularly general sensitivity to negative emotionality. Uniquely prospective and abstract in humans, anxiety serves adaptive functions in risk-assessment and harm avoidance under conditions of perceived uncertainty, particularly in evolutionarily salient domains such as social relationships and status, resource security, skills/abilities, interoception/health, and other ongoing homeostatic pressures (Nesse, 2019, 2023). While difficult to manipulate or research, proximal transitions from anxiety to low-mood likely emerge when aversive uncertainty (particularly in highly-weighted internal goals) shifts toward perceived uncontrollability, loss, reward omission, or wasted behavioral investment. Thus, while anxiogenic and depressogenic factors and domains may overlap substantially within individuals, they can differ significantly across individuals and personality profiles. Future research should focus on better delineating and comparing specific depressogenic events (Keller & Nesse, 2005) given that nomothetic risk-factors have now received disproportionally extensive empirical efforts.

### Notable Stratification Features

There remained notable overlap in predictive features across multiple mood and anxiety subtypes (Fig 4b), particularly in trait-like characteristics and recent social problems. However, certain subtypes were characterized by some distinct patterns. The high-achieving/perfectionism/depression/anxiety subtype was largely predicted by stubbornness, and school-specific worry, and social exclusion. In contrast, the ADHD/cognitive dysfunction/depression subtype was more strongly associated with oppositional-defiant features. Among females, the most predictive feature of low-mood/depression was ‘fear of going to school’—indicating partial overlap with the high-achieving/perfectionism subtype (Fig 4b). Beyond this, few features consistently differentiated males and females, though a small number of exceptions were observable.

When stratifying the sample by low versus high ALE history, predictive features suggested distinct profiles of proxies of resilience and vulnerability in children (Fig 4b). In individuals with *low* ALE exposure, predictors included recent social problems and some histrionic characteristics. In contrast, for those with *high* ALE exposure, predictive features were predominantly anxiety-related. Notably, when all personality or psychopathology measures were excluded, the strongest predictors in both groups involved the quality of social relationships. Although ALEs themselves showed only modest predictive power for mood outcomes in this sample, developmental changes occurring with severe or chronic stressful life events may nonetheless increase vulnerability to clinical depression later in life—likely through interacting genetic and social risk/protective factors (Birnie & Baram, 2025).

Lastly, we assessed ideations of suicide at all available time points (see Supplementary materials S3.4) as an additional operationalization and more severe component target of depression in parents. Overall predictive power was lower than hallmark low-mood/depression features, but across all categories ML models (particularly at basline) demonstrated moderate success (r2=0.256). Consistent features for suicidal ideation indicated were antisocial and avoidant personality styles, as well as somatic issues and relationship quality (and there changes therein), overlapping largely with overall low-mood/depression targets in parents.

### Developmental Patterns and Child-Parent Concordance

Parental psychiatric diagnosis and dimensional are well-established risk factors for a range of child outcomes. Prior results extrapolated from the ABCD dataset suggest that parent mental health may have even stronger effects on childhood internalizing symptoms than categorical adverse life events (Brieant et al, 2023). Parents can transmit risk both genetically and through environmental feedback (and vice versa) as children share genetic factors with parents and can substantially alter parents’ environments. Many parent-child psychopathology relationships appear non-specific, with aggregated parent mental-health dimensions most strongly predicting child externalizing symptoms (r2=.34), ADHD (.23), anxiety (.18–.32), and mood (.17–.23)—all moderately higher than predictions from parent mood symptoms alone (.13).

Although direct comparisons across some measures/domains were not possible, parents and children nonetheless showed similar hierarchical patterns and overlapping predictive features across most categories (Fig 3,4,5), novelly suggesting generalizability across developmental stages. Furthermore, measurement bias may be reduced in adults, as many constructs were assessed with self-report. Notably, self-reported cognitive and attention issues, anxiety, personality features, social relationships, and recent changes therein overlapped in the predictive hierarchy across parents and children (Fig 4,5,6,7). In both children and adults, recent changes in mood were most strongly predicted by concurrent changes in anxiety, social dynamics, and somatic symptoms. Stable predictors of low mood in parents included aggression, relationship volatility, anxiety, avoidance, confusion, somatic concerns, and attentional dysregulation (Fig 6b). Interestingly, in parents, self-reported ‘happiness’ (which was used as an additional operationalization of mood), was consistently harder to predict across nearly all within-category feature sets than low-mood (Fig 5), accordant with recently reported extensive domain variability in another large adult sample (Beck et al, 2025).

Family dynamics (particularly conflict related) and residential characteristics accounted for more variance in parent mood than in adolescents. In contrast, SES had comparatively limited predictive power for psychiatric outcomes in both children and adults, especially for internalizing symptoms (Fig 4,5), consistent with recent findings (Gibbons et al, 2024). Nonetheless, where SES effects were observed, they showed a strikingly linear inverse relationship with general p-factor spectra psychopathology. These associations were markedly larger for externalizing in children and internalizing dimensions in parents. This dissociation may suggest developmental changes in depressogenic factors, especially as adults may increasingly attach affective value to financial stressors and monetary goals/feedback. While higher objective SES had a modest buffering effect on adult internalizing symptoms, recent negative financial dynamics dwarfed this relationship (Fig 12), consistent with the idea that mood transitions are often dynamically accordant with changes in baseline expectations. In contrast, family conflict and the general quality of social relationships accounted for substantially more variance in mood than SES itself (Fig 3,4,7, 7Ex), suggesting that social/familial relationships exert a stronger and more developmentally stable influence on mood than SES factors alone.

In children, SES was more predictive of cognitive task and academic outcomes (Fig 3,4,5), consistent with prior findings in ABCD (Roberts et al, 2023, Brieant et al, 2023), and in other large-scale studies of psychiatric outcomes (Gibbons et al, 2024).

### Objective-Subjective Measure Discrepancy

For further domain comparison, all measures were arranged on a spectrum to the degree they might be influenced by self-report - with results showing substantial predictive differences for ADHD, anxiety, and depression (Fig 4b). Among objective measures which were likely least influenced by any report bias, number of steps and resting heart rate recorded from fitbit, waist size, being of white european descent, and grade outcomes were the strongest predictors of CBCL depression-syndrome (See supplementary materials S5.1).

Relatedly, predictors of grade outcomes themselves had discernable overlap with many psychopathology targets. However, several categories which were not strongly associated with mood did account for variation in school outcomes and cognitive tasks (Fig 3,4). ADHD measures did unsurprisingly moderately predict grade outcomes. However, ADHD counterintuitively accounted for comparatively minor variation in experimental cognitive tasks utilized in ABCD (r2=0.022) (Fig 3,3b) - whereby many such target task constructs (particularly EF-related) are often considered foundational to the disorder.

### Cognitive Paradox

Similarly, the extensive array of experimentally derived cognitive task parameters (P=88) had minimal predictive power for child anxiety and mood outcomes (r^2^=0.004), however, they were increasingly higher for Externalizing (0.016), ADHD (0.022), educational performance (0.187), and concurrently administered cognitive tasks, TB Flanker (0.235), and Reading test (0.351). Past studies have demonstrated a range of small to large effect sizes in case-control studies with formal categorical DSM disorders across a range of similar tasks examined here; however, confusingly although tasks are supposed to map onto theoretically discrete psychological functions, many such relationships are largely non-specific to categorical DSM diagnosis (termed the C-factor) (Abramovitch et at, 2021). Similarly, non-linear relationships between internalizing and experimental-task parameters have been demonstrated in ABCD (Pines et al, 2024). However, importantly, when controlling for the inattention component of ADHD in adolescents in ABCD, already minimal dimensional relationships between psychopathology and experimental task performance are completely extinguished - and even those in the top 10–15th percentile stratifications of CBCL-rated adolescent depression and anxiety (who are also low in ADHD) outperform even those low in psychopathology in several crystalized, fluid, and EF tasks, appearing in part mediated by trait perfectionism. (Roberts et al, 2023).

However, conversely, in stark comparison, parent subjective-ratings of children’s cognitive features and styles, which in theory are supposed to map onto many generally similar explanatory targets of EF and cognition, demonstrated moderate to comparatively high performance for child depression (r^2^≈0.266) (alongside other psychopathological outcomes). Similarly, parents self-reported subjective measures of cognition and attention issues were strongly predictive of adult depression (r^2^≈0.31).

This conspicuous discrepancy between *subjective* cognitive assessments and *experimental* cognitive tasks suggests fundamental differences in what measurement approaches (of often similar target psychological constructs) capture in relation to real-world functioning. Several ‘neuropsychological’ theories of depression (including facets of remission and drug efficacy) are based on results from experimental behavioral or cognitive tests in more controlled laboratory conditions, commonly in case-control studies of adults with smaller samples. Functionally decomposing ontologically relevant cognitive processes and generating experimental tasks to capture latent computations is challenging and an ongoing effort. For some widely-used psychological constructs such as ‘self-regulation’, some larger-scale studies indicate that while ranging experimental task DVs generally demonstrate robust associations with each other, their relationships with survey DVs (even when aimed at the same explanatory target) are often considerably small (Eisenberg et al, 2018). Alarmingly similar patterns are noted here within ABCD’s uniquely extensive data-acqusition effort between ‘objective’ and subjectively-rated variable types for several hallmark explanatory targets of psychiatry.

Still, for low-mood/depression, parent-rated measures of children from the SRS (T1; r2=.235), BDEFs (T3; r2=.231), and joint child/parent ratings from ABCDs Emotional Regulation survey (T3; r2=.332), all indicate that facets of emotional and cognitive styles in children, particularly those related to general domains of intrusive thinking (Visser et al, 2020), EF, processing of information, and dimensional autism features have comparatively high predictive value. Fuzzy boundaries are common here and some features were removed in primary models for concerns of circularity with the construct of low-mood among raters; however, top predictive variables within these scales included several measures of cognitive reappraisal strategies/efficacy and narrow interests (See supplementary materials S3.3). In stark contrast, the collective predictive power of the extensive range of fluid/crystalized intelligence and several paradigmatic EF cognitive tasks collected in ABCD for mood outcomes showed virtually no predictive power (Fig 4).

Unfortunately, other experimental tasks commonly used to examine facets of internalizing features (affective bias, threat conditioning, reinforcement learning) were not used here - however, they often show modest effect sizes, don’t correlate well with symptom severity, or generalize well out-of-sample (Disner et al., 2017; Marchetti et al., 2018; Beevers et al., 2019; McNally, 2019). Several past studies have indicated experimentally derivable cognitive deficits in adults across nearly all severe categorical DSM diagnosis, including mood disorders, however none appear largely specific to depression (Abramovitch et al, 2020).

Relevance of intentional content in depression and anxiety may be partly unique among explanatory targets of psychiatry (Hitchcock et al, 2022). Purported functional/psychological constructs which experimental tasks are intended to measure may still relate to important facets in the pathogenesis of aberrant anxiety or mood; however, their validity and predictive capacity in meaningful complex real-world outcomes may still be far removed. Formal computational modelling in experimental paradigms have been particularly useful for reducing ambiguity for psychological constructs which are based in natural language constraints.

Experimental-task models of internalizing, whether learning, physiological sensitivities, behavioral dispositions, or latent cognition often either involve constructs of ‘top-down’ control deficits largely researched in EF tasks (assessed here), or more bottom up conditioning/extinction, reinforcement learning (RL), and affective bias paradigms (not assessed with the exception of d-prime and performance in emotional face conditions from n-back task, which showed negligible associations with internalizing symptoms; Pearson’s *r* range = −0.038 to 0.011; see S3.3). Alongside concerns of reliability, some prevailing theoretical constraints and limitations could involve 1.) human subjects are highly context-sensitive multi-scale systems (Pessoa, 2025) capable of processing relevance realization (Jaeger et al, 2024) and representing contextual controllability during experimental tasks - the stimuli of which often have little dimensional or categorical affective relevance to idiosyncratic anxiogenic/depressogenic factors 2.) RL paradigms, which are particularly amenable to computational modelling of learning and behavior connected to reward/punishment contingencies, also contain a large degree of context-specific valuation, interoceptive input, and connections to underlying motivational and model-based goal states (Molinaro & Collins, 2023; Sinclair et al, 2023; Hewitt et al, 2025; Weber et al, 2025), including the fact that humans may disproportionately maximize for controllability over future states, compared mere reward (Munuera et al, 2023; Na et al, 2024; Ramirez-Ruiz et al, 2024) 3.) experimentally isolating aberrant modulatory or relevant facets of perception, attention, thought, affect, and behavior may be currently precluded due to their unified, coordinated, context-specific, and likely more global representations (Herz et al, 2022, Westlin et al, 2023). 4.) Even evolutionarily or normatively optimal behaviors (which can be simulated and contrasted) can still be largely susceptible to deleterious happenstance sequences (Nesse, 2022).

Potential pathways forward might include acquiring more extensive measures of idiographic functioning within and across shared important evolutionary domains (Nesse, 2019) taking subjective measures/features as capturing comparatively likely important variance (Liu & Lau, 2022; Taschereau-Dumouchel et al, 2022; Kurdi & Mandelbaum, 2023; Corneille, Gawronski, 2024), and more rigorous experimental within-subject sampling to further assess reliability across contexts (Westlin et al, 2023).

### Current State of Neuroimaging Modalities in Internalizing

Over the last 50 years there has been an explosion of interest in biological psychiatry (Insel, 2015). However there have been many recent critiques of current diagnostic classifications being best conceptualized as ‘brain’-based disorders, at least in being straightforwardly reducible to specific functional modules in neural systems (Borsboom et al, 2019; Blithikioti et al, 2025). Initial optimism was likely precipitated from the several biophysical/chemical methods able to acutely modify mood, clearly indicating a causal, necessary, albeit complex role of underlying neural states. However, challenging still is psychiatry’s capacity to generate a clear and consistent definition of what constitutes a ‘brain-disease’ (Jefferson, 2022; Aftab, 2024; Blithikioti et al, 2025), particularly for internalizing spectra, where biomarker challenges have persisted (Winter et al, 2022). Still, broad characterizations have included hypo- or hyperfunctional neural modules or systems involved in reward or threat, cognitive control, hyper-inflated or rigid predictive processing, and more complex dysregulated feedback between many such biopsychosocial elements (Wittenborn et al, 2016).

In contrast to depression and anxiety (Fig 4), results here show that grade outcomes, reading performance, and to a modest extent, ADHD and externalizing, exhibited minor to moderate associations with multimodal neuroimaging features in children (Fig 3, F4B). A recent analysis of the ABCD dataset reported a moderate negative association (R^2^ = 0.176) between white matter fractional anisotropy derived from DTI and a factor score of 18 early environmental adversity indicators (Carozza et al., 2025). In our ML ensemble stacking framework, DTI and structural neuroimaging features together yielded an even stronger relationship with the Social Vulnerability Index (used in their adversity factor score) alone (R^2^ = 0.250) (See supplementary materials S3.4), while associations with adverse life events (R^2^ = 0.009), anxiety (R^2^ = 0.001), and depression (R^2^ = 0.003) at baseline were negligible. Classification attempts targeting children in the top 5th percentile of depressive symptoms demonstrated similarly limited performance (Baseline: ROC-AUC = 0.51, F1-macro = 0.51; T2: ROC-AUC = 0.52, F1-macro = 0.55). Together, these findings highlight a salient dissociation requiring further elucidation of the capacity of neuroimaging signals to detect variability in socioeconomic adversity and cognitive performance compared to internalizing, at least within adolescents with current available neuroimaging technology.

Meta-analyses of depression and anxiety studies often find little concordance in terms of brain correlates from fMRI and EEG (Muller 2017; Sprooten 2017; Zugman 2023), perhaps because of the heterogeneity of studies and limited sample sizes therein. Driven by this realization, the neuroimaging community has moved towards large consortium studies to find more generalizable brain correlates. In the current consortium dataset along with several others, Marek and colleagues (2022) tested univariate and multivariate resting-state, structural and functional MRI predictions of continuous psychopathology measures. Results indicated correlations of *r* = −0.05 to 0.05, representing less than .25% of the variance explained. Follow-up studies using different resting-state (dynamic) measures have proven similar (Zening Fu, 2025). Similarly, Winter and colleagues (2022) analysis found no neuroimaging modality accounted for more than 2% of variance in MDD. Equivalently, results here in children with both regression and classification (See supplementary materials for classification results S3.4) using several of the current best-performing ML models in recent benchmarks (Ye et al, 2024; Hollman et al, 2025), suggest that structural, DTI, rsFMRI, and task-fMRI currently have very little predictive value for various operationalizations of depression, anxiety, and the general internalizing spectra (Fig 3,4, S3.4).

Some exceptions to this general pattern have been reported. Tse and colleagues conducted a resting-state analysis of adolescent major depression across six sites and found high AUCs (> 0.73) and significant continuous prediction (*r* = 0.13). Differences with the current study include an older, more homogeneous sample of depressed adolescents (no comorbidities), and harmonized resting-state procedures; yet the resting-state scans actually included fewer samples per individual, putatively impacting reliability. This increased performance needs to be assessed over greater follow-up durations, perhaps with sensitivity tests to cross-validation methods. A recent study in 141 adults with much richer longitudinal sampling and more formal MDD thresholds showed some promise of a potential biomarker related to salience network expansion in most individuals with MDD (Cohen’s *d* = 0.77–1.99) (Lynch et al, 2024). Interestingly, this candidate biomarker predicted depression prospectively, but was not sensitive to treatment effects nor correlate with symptom severity (other properties of the salience network, like connectivity, were responsive in these ways) These findings again require further validation - particularly as to whether it is specific to hallmark features of unipolar depression compared to more general psychopathological spectra. More extensive longitudinal intra-individual and longer durations of sampling may be required for better precision, with 1.5–2 h of high-quality fMRI data per subject suggested by (Lynch et al, 2024). Such extensive within-person data, had it been collected here, might yield a different picture of the predictive value of neuroimaging variables. Despite the challenges associated with confounding, variables such as lifetime exposure, severity, and certain inclusion criteria could also be important for functional signal discovery (Wang, et al, 2024) beyond the adolescents between 9 and 11 years of age assessed here. However, other large adult studies involving more severe clinical populations with similar measures have identified similarly limited predictive utility (Winter et al, 2022).

There may still be substantial improvements in the search for brain-based treatments and biomarkers. Biomarker replication failures have not been specific to psychiatry (Errington et al, 2021) and have been concurrent with still nascent methods in neuroscience and bewildering diagnostic variability (Fried, 2022). However, as for the larger context of ‘brain-based disorders’, we believe that our results here uphold a more complicated systems perspective, where internalizing depends on temporally dynamic, context-dependent, and multiscale biophysical signaling that is many-to-one (ie. diverse biophysical features subsumed under diverse etiological trajectories may converge onto similar hallmark internalizing outcomes and often self-reinforcing symptom dynamics).

Clinically-useful prediction will ultimately require the capacity to correctly identify and further verify input-output relationships out of sample, with common ongoing pleas towards better cross-site, longitudinal, and intra-individual validation. The apparently-limited capacity of objective biophysical measures to detect precise signals today may be due to the inherent noisiness, temporal complexity, nonlinear system interactions, self-reinforcing dynamics, and variability in causes, symptoms, and behaviors in depression. Scientific progress on proximal temporal transitions is further precluded by the challenges in meaningfully experimentally manipulating aversive states or idiosyncratic depressogenic and anxiogenic factors.

Given the comparatively high and increasing rates of anxiety and depression compared to other mental health problems in the general population (WHO, 2023), a more general elucidation as to why evolution left so many biopsychosocial control systems vulnerable towards distress and dysregulation in the first place is warranted (Nesse, 2019). While prone to tradeoffs, myriad environmental mismatches, happenstance, organically complex failure-modes (Nesse et at, 2025), certain aspects of what is currently termed ‘psychopathology’- regarding both externalizing and internalizing - could have provided several evolutionary advantages within certain ancestral contexts (Dalley et al, 2011; Nesse, 2023), particularly under cognitive resource constraints (Bari & Gershman, 2024)

Developing novel more granular idiographic measures could benefit and better bridge nomothetic domains in psychiatric research (Nesse, 2019), further synergistic with more intensive within-person longitudinal collection (Hamaker, 2025) and higher temporal resolution data (including that collected in some ongoing experiments; Fried et al, 2023).

## Limitations:

Uniquely, many null results were reported corresponding to the conceptual aim of the research. Our methods were in part chosen to overcome issues inherent to data-acquisition and non-controlled experimental designs. Nevertheless, the biological, psychological, social, and environmental range of data collected in ABCD is currently unparalleled at this sample size. However, there are many inherent challenges for acquisition at this scale, including biospecimen data (Uban et al, 2018). Further, insufficient effort and careless responding on survey and task data appear common in research, particularly noticeable among unpaid undergraduates and online data platforms (Huang et al, 2012; Arias et al, 2020), and could have been present in the current dataset.

As a precautionary note, ‘hierarchy’ is used here in a largely statistical sense and should *not* be equated with theoretical models for many reasons (Fried, 2020). Some of the underperforming categories above (Fig 3,4,5) still 1.) clearly have latent variables which were not acquired or are very difficult to measure even in theory and 2.) some measures exhibiting very minor albeit consistent associations and temporal trends. Theoretically likely is that some categorical domains assesed will evolve in importance across development, some of which were emphasized in results such as SES proxies. Still, available similar measures/categories used in parent analysis (Fig 5) demonstrated notable convergence of many several predictive features and categories, indicating some novel developmental generalizability to results.

Among the several possible sources of measurement bias, the primary reliance on parent-rated measures for many predictors and outcomes was a notable limitation emphasized throughout. Confusion and concerns of how to reliably and effectively measure internal subjective affective and mood states are ongoing in psychiatry, particularly as such states can difficult to proximally assess, can contain physiological variability, raters may be individually or culturally bias, and there is no clear veridical indication of accuracy as compared to other psychological constructs such as object recognition (Roberts et al, 2020; Fried et al, 2022; Lindquist et al, 2022; Taschereau-Dumouchel et al, 2022; Rust, 2025). The parent-child ratings in the CBCL were partly developed around DSM-items and has shown moderate inter-rater reliability (Achenbach, 2001). Extensive attempts were made to remove potentially circular or tautological variables from predictor sets in ML models for all psychiatric targets. However some constructs among parent-raters may have been difficult to conceptually distinguish between (particularly, anhedonia, worry, stubbornness or impulsivity, rule-breaking, etc), particularly in the dimensional range.

For our primary mood outcome (Fig 4), we attempted to remove traditional secondary features of depression related to sleep, appetite, and cognitive difficulties - so as to focus on the hallmark features of low-mood, particularly the inability experience pleasure, chronic sadness, and suicidal ideations. However, for validation towards pathosuggestive symptom interactions, we also analyzed CBCL depression syndrome scores within primary CLPN and BMM models, as well as ML models which can be seen in supplementary materials. Many still believe that clinical MDD is more than an amplification of sub-clinical low-mood and likely involves a tipping point in severity and the self-reinforcing elements of secondary features in the diagnosis. Thus, we also assessed the top 10th and 5th percentiles of low-mood features from the CBCL (Fig 6) as well as the KSADs (see supplement S3.4). While the KSADs is often considered to provide a more structured method for assessing psychiatric diagnosis, ABCD employed a computerized version of the KSADS with research assistant support differing from more traditional clinician-administered interviews and only of a subset of children were examined (Karcher & Barch, 2021). Further, KSADs assessment was also only administered at 2 of the 5 time points and contained much fewer observations, with only 59 meeting the formal MDD diagnosis, 232 for single items of depressed mood, and 447 for single item anhedonia. Thus, despite the far-reduced range of predictors, we ran classification models for validation purposes.

Despite the growing rates of internalizing issues often noted in younger generations, the age range of the ABCD cohort (9–14) is lower than the average age of onset of clinical depression (Solmi et al, 2022). Available parent data was included to partly circumvent having to make insufficient generalizations. 90% of parent data was collected from mothers. While there was a considerable amount of child-parent overlap, there were also important developmental differences which were emphasized (Fig 3,5,6,7,8).

Despite ACBD’s extensive variable acquisition effort and our adjunctive analysis of cross-lagged effects to possibly infer causal realtionships, yearly intervals of data extraction and some different variables being collected at different time points in ABCD increase the possibility of potentially important missing information within or between time points (Hamaker, 2025). Intentional domains and proximal depressogenic factors are difficult to acquire or assess but future research efforts are warranted given their importance in the commonly episodic nature of depressive states. Here, several methodological approaches were utilized in attempts to address this problem, including creating new variables for the delta of important variables from the last time point, estimation of clinically relevant temporal changes, and implementing longitudinal statistical models where possible with more coarse-grained CBCL syndrome scores. Despite indicating some possible causal features and interactions, delineating the direction and structure of causal dynamics is difficult to infer from statistical models alone.

## Conclusion:

Coarse-graining complex multi-scale systems involved in psychopathology, which appear to consist of difficult to measure variable idiographic feedback loops and etiological trajectories, has been a challenging endeavour in psychiatry. Findings from this large longitudinal dataset, examining a comprehensive range of existing biopsychosocial measures, suggest that robust prediction of psychopathology (including anxiety and depression) is still achievable, largely via interactions of specific psycho-social traits and dynamic processes. Demonstrated here, these include negative dynamics in social relationships, somatic issues, family conflict, and ongoing ineffectual goal pursuits and more stable, trait-like characteristics, particularly cognitive rigidity, interpersonal antagonism, and an elevated sensitivity to social feedback. Providing both challenges and insights to current diagnostic classification schemes, many such features and others appear highly predictive of transdiagnostic mental health problems. Moreover, dysregulated attentional systems emerge as a central and longitudinal predictor in the nexus of broader psychopathological dimensions - appearing both as a cause and effect of ranging mental health challenges across the lifespan. Results further illustrate that many dimensions, particularly internalizing, are still minimally predicted by many *current* biophysical, experimental tasks, and several more objective-type measures. Often in smaller samples, while some past studies have demonstrated statistically significant differences, research has also contained mixed, sometimes contradictory, and ranging effect sizes for biophysical or task measures in depression, anxiety, and ADHD. Noting potentially important demographic and clinical differences, some results may have been overstated in their validity, diagnostic specificity, and explanatory power. Methods, technology, and clinical applications of non-invasive neuroimaging are a rapidly progressing endeavor which could facilitate better prediction, explanation, and control at some point. However, our conclusions affirm the emerging sentiment that hard reductionism is largely inadequate at this time to ground high-level psychological features and interactions that characterize psychopathology, particularly internalizing. Still, hard-reductionism here should not be conflated with attempts to better compress and formalize interacting elements of dynamic systems generating psychopathological states or traits. Dimensional and severe clinical conditions may likely involve a complex mix of nomothetic, idiographic, longitudinal, proximal, dynamic self-reinforcing, and possibly highly biologically heterogeneous factors. Ultimately, collective interventions could be effective, but idiographic complexity could make them difficult or impossible to implement at scale, and thus modifiable factors which are central to individual pathways are constructive targets in the near term.

## Methods:

### Methodological Trajectory

Specific details of our analytic pipeline are outlined below. However, our first primary methodological aim was to organize a wide, complex range of biological, psychological, social, and environmental measures. Some measures were collected at different time points from the 5 years of data acquired. Secondly, to overcome some of the tradeoffs and limitations of purely relying on methodological approaches for data-driven high-dimensional prediction, we employed several adjunctive and synergistic analytic methods to maximize interpretability, uncertainty estimation, and provide longitudinal and *potentially* causal insights.

1. Regarding our more exploratory high-dimensional predictive models, for simplicity, we outline the broader categories for which all extracted measures were grouped for adolescent and parent analysis in Figure 1. Figure 2 then overviews all extracted measures (minus neuroimaging) which were used in subsequent predictive models for one of the larger acqusition time points and displays their associative structure in an edge-bundling diagram. Primary models for high-dimensional prediction (Fig 3–6) employed machine learning using an ensemble stacking approach (combining TABPFN, CATBoost, XGBoost, and Random Forests with a linear meta-learner). These models provide several key advantages over traditional correlational approaches, including the ability to capture non-linear relationships, high-dimensional interactions, and in the case of TABPFN - transformer-based in-context learning to approximate Bayesian posterior distributions and uncertainty estimates (Hollman et al, 2025). Our stacking method further leverages complementary strengths of each model to improve generalizability and reduce overfitting with diverse high-dimensional predictors. Target outcomes in predictive models included several operationalizations of low-mood/depression (see below; Fig 4b), other psychopathological features (Anxiety, ADHD, Externalizing, Social Problems), and some cognitive outcomes (NIH Toolbox (TB) Reading, TB Flanker, Grade Outcomes) (Fig 3). Alternative targets were used to contrast similarities and differences of predictive features, and provide more objective measures which relied less on subjective self-report ratings. For each, we assessed the comparative predictive power and interactions both within categories and across the full-range of biopsychosocial domains/measures (Fig 1).
2. Regarding subsequent complementary analysis, to maximize interpretability and coherence, we employed several methods for better assessing more theoretically inspired coarse-grained and longitudinal data. For available and theoretically relevant longitudinal data, autoregressive cross-lagged panel network (CLPN) models (Fig 8) were utilized to explore undirected and bidirectional relationships as well as potential causal developmental pathways between psychopathological dimensions over time, without the designation of any specific outcome variables. This approach allowed us to examine how different psychopathological features potentially influence each other across development while accounting for temporal stability of each construct. We further supplemented these network analyses with Bayesian mixed models (BMM) (Fig 7), which offer several advantages for longitudinal data analysis, including robust handling of missing data, more accurate uncertainty estimation, and the ability to model both population-level effects and individual-level variability simultaneously while controlling for covariates. BMMs provided a valuable contrast to CLPNs given they have a directed specific target outcome, also providing uncertainty quantification in parameter estimates. Lastly, ANCOVAs were used for examining stratified categories such as family income brackets.

### Data Collection and Sample

Given the wide scope of data collected in the ABCD dataset (www.abcdstudy.org) many extraction, organization, pre-processing procedures, and subsequent statistical modelling choices were implemented to compare wide-ranging biopsychosocial domains. Data from adolescents (N=~11,000; ages 9–14) was collected across five yearly-spaced time points between September 1, 2016 to January 15, 2022. Further, while data acquisition in the ABCD consortium was primarily oriented around adolescents, there was also extensive data collected from parents (mostly mothers ~90%) which we also extracted, organized, analyzed, and compared (when available), which increased the sample size to N=~23,760. However, some measures and broader categorical domains were not available for parents making some comparisons impossible. After data cleaning procedures and exclusion of variables with more than 20% missing values, the final sample for primary ML analysis consisted of 15,366 combined adolescents and parents subjects. Adjunctive longitudinal analyses relied on variables which were acquired only across all time-points (such as lower-dimensional syndrome scores from the CBCL).

### Variable Selection Into Categories and Feature Engineering for High-Dimensional ML Models

Approximately ~1,078 unique variables were selected or created from 69 different scales available in ABCD (See supplementary materials) based on three criteria: 1.) potential theoretical relevance to psychopathology 2.) most informative for comparing hierarchical differences and informing subsequent research and 3.) prior empirical evidence of association. Figure 1 outlines the final categories used for within and across category comparisons. The complete variable selection, number of variables in each category, scales used for extraction, processing procedures, and extra information about hierarchical feature importance within categories are provided in the supplementary materials; to assess the stability of conclusions across these categorization schemes, further and alternative categorizations are also explored in other analyses, as described below.

Our categorization scheme was novel though it contained some similarity to the ABCD consortium’s original ontology or arrangement of categorical domains (https://abcdstudy.org/about/). Specific variables were chosen from all scales if they had any possible theoretical relevance or prior empirical evidence related to internalizing spectra or general psychopathology. Similarly, our feature-engineering categorization of variables was based on theoretically relevant domains (See Figure 1) that are commonly assessed independently in prior studies and/or those that might be the most informative for comparing hierarchical differences and informing future research.

Range of available variables was organized into 28 primary categories for children and 20 for adults. The grouping scheme here contained several paradigmatically biological, psychological, and social domains including a range of neuroimaging modalities, cognitive task outcomes, personality and co-occurring psychopathological features, medical/somatic problems, technology use, quality of social relationships, family dynamics, socioeconomic status, and several others. (see Figure 1 for categories and Supplement for all specific variables at each time point)

During feature engineering, we attempted to acquire a high-dimensional range of measures/features, particularly focusing on parcellating along a biological, psychological, social, and environmental gradient (Fig 1). We removed variables that 1.) overlapped theoretically in their constructs 2.) would be difficult for raters to distinguish and 3.) or already subsumed in predictive targets, ensuring distinct and meaningful predictors. With the exception of neuroimaging and genetic features, we chose not to apply dimensionality reduction to preserve high-dimensional feature sets for novel analysis.

### Further Fractionation of Ontologically Relevant Categories

We further attempted to parcellate variables which were more ‘objective’ (ex. neuroimaging, cognitive tasks, genetic PCS, etc), subjective parent-rated, and child-rated measures (Fig 8). This allowed us to differentially compare across ‘objective’ and self-report domains using the analytic machine learning (ML) methodology described below.

### Machine Learning Pipeline

After feature-engineering many theoretically relevant categories, we assessed high-dimensional predictive relationships using a variety of the best performing ML models in recent benchmarks (Ye et al, 2024; Hollman et al, 2025) both within and across selected categories (Fig1). We closely followed recent consensus-based recommendations for using machine learning methods in science (Kapoor et al., 2024; see supplement for a summary checklist of guidelines and rationale).

Several tree-based algorithms were selected for their ability to capture complex non-linear relationships and interaction effects, and their ability to readily handle mixed variable types (e.g., categorical and continuous), making them well-suited for our wide-ranging feature engineering.

To analyze high-dimensional predictive relationships, we employed a multi-step machine learning (ML) framework utilizing an ensemble stacking method that combined multiple models to enhance predictive power, stability, and generalization while minimizing variance and overfitting. This approach capitalizes on the complementary strengths of the different representational formats in the models described below.

TabPFN, a novel transformer-based fitted network, was incorporated for its ability to leverage Bayesian reasoning over tabular data distributions, allowing for better inference, adaptive learning, and built-in uncertainty quantification, demonstrating comparatively high predictive performance with tabular data with similar samples and parameter sizes (Hollman et al., 2025) that were used in our analysis. CATBoost, is a gradient-boosted decision tree (GBDT) model which commonly performs well in mixed-type data sets (ref) and was chosen for its robust handling of categorical variables and ability to reduce overfitting to enhance predictive accuracy in structured datasets. XGBoost, another highly efficient GBDT framework, was included for its strong regularization techniques, flexible objective functions, and scalability, particularly useful for handling missing values and high-dimensional data with complex interactions. Random Forests, a more traditional ensemble utilizing decision trees, known for robustness to overfitting and noise, making it useful for feature selection, estimating feature importance, and handling heterogeneous data distributions. Lastly, a Linear Regression Meta-Learner (or Logistic for classification) was employed to aggregate base model predictions, refining generalization by reducing bias and variance from individual models, ensuring that final predictions were both robust and interpretable. This multi-modal approach ensured that the strengths of each model were leveraged appropriately, balancing predictive accuracy, generalizability, and interpretability with mixed data-types across biopsychosocial domains. All models were trained using nested cross-validation (5 outer folds, 3 inner folds) to prevent overfitting and obtain unbiased estimates of model performance.

Feature importance rankings were jointly derived from SHapley Additive exPlanations (SHAP) values, permutation importance, and consensus ensemble importance scores (see Supplementary Materials for all SHAP values for each model). Given the clinical significance of prediction uncertainty, we also utilized prediction intervals from TabPFN and added notable features from categories into Bayesian Mixed Models, allowing for a high degree of confidence estimation. Simpler lasso and ridge models were also used for validation and comparison to our ensemble stacking ML approach, but did not perform as well.

### Extracted and Created Psychopathology Measures

The Child Behavior Checklist (CBCL; Achenbach & Rescorla, 2001) served as a source of both predictor and outcome variables in this study. CBCL was selected due to its comprehensive assessment of a wide-range of behavioral, emotional, and psychopathological problems. All measures from the CBCL are rated by parents. Critically for modelling purposes, parent ratings of children were one of the few scales that was given across all five current time points in the ABCD dataset. Relevant items from the Behavior Rating Inventory of Executive Function (BRIEF) were also included. A slightly different parent self-reported scale of psychopathology, Parent Adult Self Report Raw Scores Aseba (ASR) was used in ABCD and also utilized here, but was only available at 3 timepoints, which were separated by 2 years, compared to child CBCL, which was collected at all time points separated by 1 year.

Both child and parent CBCL scales provide an extended range of psychopathological features, lower-dimensional composite scores based on factor analysis, as well as other more dimensional personality features (which could be deemed as psychopathological at extremes). High and low-dimensional CBCL measures, including alternative target outcomes for comparison (anxiety, adhd, externalizing), were used in the several statistical modeling strategies applied within the central aims of the paper.

### Operationalizations of Depression

Recognizing the notorious variability of depression and corresponding measurements (Fried et al, 2022), we created several operationalizations using CBCL measures to attempt to address this problem as well as enhance validation. Firstly, to focus more directly on hallmark depressive features, we modified the standard CBCL depression composite by excluding items related to sleep, appetite, and somatic complaints, which may be secondary to or bidirectional with mood disturbances. This allowed us to assess secondary features as predictors themselves (comparing to other outcomes), and more directly focus on hallmark features of low-mood and sometimes depression. Namely, measures likely related to more *chronic* anhedonia, dysthymia, and sadness, proxied by the parent-rated CBCL items were rated as 0 = None of the time, 1 = Some of the time, and 2 = Most of the time. Further, exclusion of secondary depression features was done as it is not clear how central or unique some of these features are to depression compared to other psychopathological dimensions. Similarly, as parent outcomes were also measured, we only used the self-report measure of low-mood/depression as a target outcome. However, for validation purposes, we also provide depression composite/syndrome scores for predictive models in CLPNS, BMMs, and the supplement for ML - which for the latter had very modest differences between hierarchical prediction capacities of categories assessed.

As a cautionary note for *some* of the models, the CBCL may represent dimensional (though likely chronic based on psychometric orientation) *low-mood* more than severe states of clinical depression. Dimensional measures were pragmatic due to 1.) more substantial coverage across the full sample and all time points and 2.) some of the methodological and theoretical limitations of binary classifications in psychiatric research (Scott & Nelson, 2024). Still, despite limitations inherent in the data acquisition, ML classification was performed for possible insights into more severe cases when possible (described below).

Given this and the well-known heterogeneity and episodic nature of depression, we created multiple other operational variables to further assess theoretical and statistical differences and provide extensive validation. Thus we operationalized and use the generated variables for low-mood/depression in the following way:

1. **Symptom dimensional outcomes:** dimensional features were utilized primarily in regression models to assess hallmark features of low-mood. Composite and syndrome depression scores from CBCL including secondary features are also available in the supplement.
2. **Recent clinically relevant onset from below baseline or dimensional change:** We generated new variables with the Reliable Change Index (RCI) (Jacobson & Truax, 1992) at a threshold of 2.3, capturing clinically significant increases from low symptom levels from the last time point (one year prior). Dimensional deltas from last time point were also acquired and assessed.
3. **Severe cases:** CBCL percentiles (top 5th and 10th) of low-mood/depression were also assessed using classification models to capture the upper range of symptom severity. Suicidal dimensions were also assesed and are available in the supplement.

Validation was performed on the KSADS-5 more formal diagnostic criteria and is available in the supplement. KSADS was administered by research assistants at only 2 of the 5 time points with a subset of children (Karcher & Barch, 2021), containing much fewer counts, with only 59 meeting the formal MDD diagnosis, 232 for single items of depressed mood, and 447 for single item anhedonia. Given class-imbalance issues, we ran ML ensemble models for single item anhedonia and depressed mood.

### Alternative Target Prediction Comparison Points

ML ensemble models were subsequently run with alternative target outcomes to illustrate predictive similarities and contrasts across biopsychosocial measurements. Alternative target outcomes included other psychopathology (anxiety, ADHD, externalizing) dimensions also from the CBCL, educational outcomes based on school grades, as well as reading and flanker cognitive task outcomes from the NIH Toolbox (https://wiki.abcdstudy.org/release-notes/non-imaging/neurocognition.html).

### Stratification of High and Low ALE Individuals

To address the established impact of adverse life events (ALEs) on depression, we stratified individuals based on their ALE history. This stratification aimed to uncover distinct predictive patterns associated with resilience (protective factors in high-ALE individuals) and vulnerability (risk factors in low-ALE individuals). We did this by creating new binary variables under the following criterion

### Low ALE

The final criteria was set for having 0 reported ALES, not being in the 20% most disadvantaged group from the area deprivation index, parents having not had any past diagnosis of MDD, current drug problems, or high in aggression. This resulted in a remaining sample of 2,075.

### High and Severe ALE

For the high ALE group, individuals were classified if their adverse life events score exceeded 1.5 standard deviations above the median. Additionally, a subset labeled as high-risk or ‘severe ALE’ was classified if this also included high parental aggression (above the 80th percentile at any time point on parent self-rated aggression) or their parent had any past diagnosis of MDD via the KSADs assessment.

To assess possible risk and protective factors we ran full within and across category ML models in both High and Low ALE stratifications. As other psychopathological features typically dominated predictive features we excluded all such variables and ran in a different model for supplement.

### Modeling Challenges and Attempted Solutions

Different variables were collected at different time points in the ABCD study, affecting ability of some target outcomes comparisons and longitudinal modeling capabilities. To address this challenge, higher-dimensional ML models were run independently at each timepoint with available variables in our selected categorization scheme. High-dimensional predictive models for alternative target outcomes (Fig 3) were only derived at time point 2 as it contained the largest number of measurements from different categories for comparisons. Feature sets and categories not collected within specific data acquisition periods or which contained an insufficient number of variables to support extrapolation were dropped at respective time points. (See supplementary materials for variables used at each time point).

Though limited by variables that were collected consistently across timepoints, delta calculations were generated for many theoretically relevant variables (computed as change scores from the last time point) to attempt to capture how significant changes between time points affected target outcomes in ML models. Many were derived from the CBCL due to consistent acquisition across time points. However, other delta calculations were done on changes in grade performance, school problems, family conflict, and ALES on available time points.

Autoregressive cross-lagged panel models (described below) were also used for longitudinal analysis and possible infer causal relationships between lower-dimensional features from the CBCL.

### Experimentally Derived and Self-Report Cognitive Parameters

The ABCD consortium collected data from an extensive range of commonly used cognitive assessments in psychology and neuroscience research (Casey et al, 2018; Karcher et al, 2021). Core metrics such as accuracy, reaction time, and d-prime (when applicable) were extracted from all task domains across all time points. In addition to traditional measures of cognitive control, we incorporated individual level parameters identifying distinct components of dynamic cognitive control, including proactive, reactive, and attentional mechanisms, obtained via the validated PRAD model (Mistry et al., 2024). Parameter estimates obtained based on a hierarchical Bayesian implementation in JAGS, improved upon traditional measures like SSRT by overcoming limitations of traditional models, such as the inability to provide robust measurements when assumptions like context and stochastic independence are violated, the inability to account for intra-individual variability, and the inability to capture trial-level dynamics and sequential dependencies across proactive, reactive, and attentional dimensions of inhibitory control.

In contrast, we also utilized several parent-to-child self-report scales that broadly captured cognitive and regulatory styles, including the Socioeconomic Environment Scale (SES), the Barkley Deficits in Executive Functioning Scale (BDEFS), and measures from the Emotion Regulation Questionnaire (ERQ). These scales provided more trait-like, dispositional indices of cognitive control and emotional reactivity, offering further indications of child cognition beyond performance-based task measures.

### Neuroimaging Data Processing

Neurobiological features were derived from the ABCD dataset at two available timepoints across all four modalities: diffusion-weighted imaging, structural MRI, resting-state MRI, and task-based fMRI (including MID, N-BACK, and SST tasks). We followed previously established feature extraction procedures for each modality in the ABCD dataset (Xiang et al., 2022; Wu et al., 2023; Gracia-Tabuenca et al., 2024). The Desikan–Killiany atlas was used for cortical ROIs and Freesurfer’s ASEG was employed for subcortical segmentation. For each modality, after excluding columns and rows with >20% missing data, any remaining missing values were imputed using the Multivariate Imputation by Chained Equations (MICE) algorithm. For dimensionality reduction, we applied principal component analysis (PCA) to reduce the data from each modality to 75 components (Sripada et al, 2020), capturing approximately 80% or more of the variance (See supplementary materials), which reduced complexity and facilitated a common framework for cross-modal comparisons and integrated predictive multivariate analyses.

### Structural MRI

High-resolution T1- and T2-weighted images (1 mm^3^ isotropic) were processed using FreeSurfer v5.3. Preprocessing included gradient nonlinearity correction, B1-bias field correction for intensity inhomogeneity, and registration to an isotropic template. Cortical reconstruction provided measures of cortical thickness, surface area, gray matter volume, and sulcal depth with parcellation based on the Desikan–Killiany atlas (Desikan et al., 2006; Fischl et al., 2002). Subcortical volumes were also extracted.

### Diffusion Imaging

DTI data (1.7 mm^3^ isotropic; 96 directions at b-values 0, 500, 1000, 2000, and 3000) underwent corrections for eddy currents, head motion, susceptibility artifacts, and gradient nonlinearity. White matter tracts were segmented using AtlasTrack (Hagler et al., 2009), and standard DTI metrics including fractional anisotropy, mean diffusivity, longitudinal diffusivity, transverse diffusivity, intensities, and contrasts were extracted from the Desikan–Killiany parcellation. We also extracted RSI (e.g. restricted normalized isotropic (N0)) data features.

### Functional MRI

MRI data were acquired using a gradient-echo EPI sequence (2.4 mm^3^ isotropic; TR = 800 ms, TE = 30 ms, multiband factor = 6). *Resting*-*state fMRI*: As described in (Casey et al, 2018), participants completed four 5-minute resting runs while fixating on a crosshair. Preprocessing steps included slice-timing correction, realignment, normalization, nuisance regression, and temporal band-pass filtering (0.009–0.08 Hz). Functional connectivity matrices were generated by computing Fisher-transformed Pearson correlations from average time series of 352 ROIs (333 cortical ROIs defined by Gordon et al., 2016 and 19 subcortical ROIs from FreeSurfer segmentation), after excluding time points with framewise displacement (FD) > 0.2 mm.

#### Task-based fMRI:

Following the procedures in (Gracia-Tabuenca et al., 2024), task-fMRI data from the Monetary Incentive Delay (MID), Stop Signal (SST), and emotional N-back tasks were processed analogously. For task-based fMRI specifically, head motion was regressed out and outlier volumes removed; in addition, sensitivity analyses were performed on the top quartile of low-motion individuals. Task contrasts were estimated using a general linear model that incorporated baseline and quadratic trends along with motion parameters (with motion covariates band-pass filtered at 0.31–0.43 Hz using an infinite impulse response filter); volumes with FD > 0.9 mm were censored. Cortical (Desikan-Killiany) and subcortical (Freesurfer) regions were examined.

### Genetic Ancestry Principal Components

We incorporated genetic ancestry principal components (PCs) in predictive models as previously derived from the ABCD genetic dataset using PC-AiR, a method specifically designed to capture population structure while accounting for relatedness within diverse cohorts (Fan et al, 2023).

Genetic PCs were grouped with self-reports of ethnicity in Within-Category ML models to potentially capture any important variation in ancestral genetics or sociological features. Prior work has indicated the importance of examining associations with ancestral structure as it may create a form of cryptic relatedness which can otherwise bias associations amongst multimodal features in diverse samples like ABCD (e.g., Huang et al, 2022).

### Brief Overview of Feature Sets included in Other Categories

Several other measurement domains were used which have been outlined prior (Karcher & Barch, 2021). Our full range of measurements included in each category at each time point in predictive analysis can be seen in the supplement. However, as a brief overview, other arranged categories used in predictive models include:

### Medical/Somatic Problems

Features related to frequency or intensity of symptoms such as headaches, stomachaches, and other physical discomforts.

### Family Dynamics & Parenting

Encompasses indicators of conflict, communication, cohesion, organization, marital status, number of sisters and brothers, and other features of parenting-styles as reported by children and parents.

### SES & Mobility

Includes socio-economic indicators such as education, joint parent income, recent financial dynamics, and others related to financial stressors or instability (e.g., difficulties with paying bills or rent).

### Religion

Few available though potentially important features were extracted here, including self-reported religious affiliation, worship service attendance, and perceived importance of faith. This category was used as a rough proxy in parent outcomes given some common and likely overlap in religious affiliation.

### Family Drug Use

Ranging parent-rated measures on use or past use of a wide-range of drugs, including exposures during the prenatal period.

### Diet/Nutrition

Parent estimations of child dietary habits and nutritional intake - including consumption of fruits and vegetables, intake of proteins, sugars, fats, etc. Also one measure was simply a subjective measure of overall bad diet in children.

### Adverse Life Events

Indices of stressful and traumatic exposures reported by children and caregivers, including global counts of negative events and subjective-ratings of their impact. Features also include specific incidents such as accidents (e.g., car crashes, fires, injuries, and natural disasters), exposures to violence and social unrest (e.g., terrorism, war-related deaths, stabbing or shooting incidents), interpersonal threats and abuse (including various forms of sexual and physical abuse), and sudden family losses. Includes both child and parent ratings.

### Social Relationship Quality

Indices of children’s peer interactions and friendship quality. It also assesses peer acceptance versus rejection (for instance, feelings of exclusion or being left out), negative interactions (like bullying, rumor-spreading, and overt aggression), and perceptions of discrimination from teachers, other adults, or peers. Includes both child and parent ratings.

### Cross-Lagged Panel Network Model

Given many of the inherent difficulties of disentangling cause and effect statistically (Wysocki et al. 2021) in adolescent psychopathology and depression, particularly when variables can both influence and be influenced by one another over time, we employed cross-lagged panel network modeling to further probe potential reciprocal relationships. This partly circumvents the inability of our ML methods to model temporal patterns due to different data collection protocols (ref) across time points.

Specifically, and by way of contrast to static network or path models, cross-lagged panel networks capture how a variable at an earlier time (e.g., T0) may predict changes in both itself and other variables at subsequent time points (T1, T2, etc.). This is especially important in internalizing disorders like depression, where features such as co-occurring psychopathology, social relationship quality, and sleep disturbances can simultaneously act as both causes and consequences of low mood. Although cross-lagged panel models cannot establish definitive causality, they provide a richer framework for examining whether changes in one variable precede shifts in another while controlling for prior levels of both variables.

Moreover, representing these temporal relationships as a network clarifies which variables are most central in driving subsequent changes and reveals potentially crucial feedback pathways. Accordingly, our cross-lagged panel network analyses serve as a bridge between high-dimensional machine learning models and traditional longitudinal methods, offering more nuanced insights into how key biopsychosocial factors evolve and interact over time.

Feature choices were largely reduced given fewer variables were collected across all times. Thus, variables were selected if 1.) had theoretical or causal relevance 2.) were low-dimensional or sparse features already demonstrating predictive power to reduce model complexity and 3.) had available data across time points. CBCL variables were collected at all time points and composite CBCL measures were used in light of their higher and more coarse-grained predictive power, having been derived from previous factor analysis (ref). This allowed us to approximate possible directional effects and feedback loops that cross-sectional or simple longitudinal analyses are often ill-positioned to estimate.

In CLPN implementation, network models were estimated using LASSO regression (cv.glmnet package; Friedman et al., 2010) employing 10-fold cross-validation to select the optimal regularization parameter (lambda.min), and standardization of variables prior to analysis. Edge weights represent regression coefficients estimated via LASSO, with edges below 0.15 set to a minimum visualization threshold of 0.15 to enhance readability. To better visualize typically weaker cross-lagged relationships compared to autoregressive effects, we applied different visual multipliers to cross-lagged (×8) versus autoregressive (×2) pathways. Network visualization and centrality analyses were conducted using the qgraph package (Epskamp et al, 2017). We calculated multiple centrality metrics including OutStrength (sum of outgoing edge weights, representing a node’s influence on other variables over time), InStrength (sum of incoming edge weights, indicating susceptibility to influence from other variables), as well as Betweenness and Closeness centrality. To ensure robustness of our network findings, we implemented a case-dropping bootstrap procedure (nBoots = 1000) to assess the stability of edge weights and centrality indices (Epskamp et al, 2018). This approach systematically drops random subsets (10%) of the sample and recalculates network parameters, allowing us to quantify the reliability of our network metrics under sampling variation. Consistent spring layout was maintained across all time transitions to facilitate visual comparison of network evolution across development.

Comprehensive stability coefficients, correlation stability plots, and detailed centrality metrics are provided in the supplementary materials, offering transparent evaluation of the network’s statistical reliability.

### Bayesian Mixed Models

To complement our machine learning approaches and examine relationships with enhanced interpretability, we employed Bayesian hierarchical mixed models. These models offer several methodological advantages for our research questions and also provide a useful contrast to the autoregressive cross-lag network panel models - which do not have a primary target outcome. First, they provide robust handling of missing data, which is particularly valuable given the varying data collection protocols across time points in the ABCD study. Second, Bayesian methods offer more comprehensive uncertainty quantification through posterior distributions rather than point estimates alone, allowing us to assess the reliability of effects across predictors.

The hierarchical structure of these models enabled us to simultaneously examine population-level effects while accounting for individual-level variability through random intercepts for subjects. We modeled relationships between depression syndrome-composite outcomes and three sets of predictors: (1) change variables (deltas) calculated between consecutive time points, (2) other psychopathological composites from the CBCL, and (3) non-psychopathological variables on depression composite scores - wherein other any CBCL measures were excluded to avoid circularity and also provide insight as to the effects of more objective and non self-report measures.

Models were estimated using weakly informative priors to stabilize parameter estimation without unduly influencing posterior distributions. We implemented 4 MCMC chains with 8,000 iterations (including 2,000 warmup) and assessed convergence using Rhat values (<1.01) and effective sample sizes. Results are presented as standardized regression coefficients with 89% and 95% highest density intervals in Figure 7, allowing for direct comparison of effect sizes across predictors and between child and parent models. Overview statistics for each model are reported in the BMM supplementary materials, as well as ADHD as an alternative outcome.

### ANCOVA Models

Our primary outcomes were parent self-report questionnaires evaluating current levels of internalizing and externalizing psychopathology in themselves taken from ABCD Parent Adult Self Report Scores Aseba (ASR) and their offspring ABCD Parent-Child Behavior Checklist Scores Aseba (CBCL). Sum scores of internalizing and externalizing were standardized and treated as continuous/scalar variables. The three predictors of interest were objective household income (ABCD parent demographics survey), recent negative change in finances (taken from the ABCD parent life events scale), and frequent family conflict (taken from the ABCD Parent Family Environment Scale-Family Conflict Subscale). Household income was divided into 7 ordinal levels (in USD): 16–25k, 25–35k, 35–50k, 50–75k, 75–100k, 100–200k, and 200k+. We thus applied 4 separate one-way ANCOVA models assessing the differential relationships among household income on familial psychopathology: household income influence on parental internalizing (1) and externalizing (2), and household income influence on child internalizing (3) and externalizing (4). Upon examining significance of any omnibus test, we applied Tukey’s post hoc tests to interrogate household income level-specific differences. All ANCOVA models controlled for parental age, marital status, education level, and sex. Following transformation of all skewed variables (absolute skew > 1), data was assessed for normality via visual inspection in addition to the application of Shapiro-Wilk tests, homogeneity of variance between variables with Levene’s tests, and outliers (defined as data greater than twice the interquartile range, above the third or below the first quartile).

In a confirmatory manner, significant effects of house income on psychopathology were further tested against recent negative income dynamics (i.e., income loss from the last year only). The robustness of the respective effects of household income and recent income dynamics on different psychopathology subtypes in both groups was compared via effect size in partial eta squared (η-p2). Covariates used in these ANCOVA models- which were treated as secondary outcomes of interest- included relevant demographic information such as parental age, parental marital status, and parental educational level, while sex (of either parent or child) was treated as a fixed factor in order to identify possible income*sex interaction effects on psychopathological dimensions.

We further implemented two types of subsidiary ANCOVA analyses controlling for relevant demographics: 1) for both parents and children, we assessed the influence of family conflict (dichotomous variable: infrequent family conflict= 0, frequent family conflict=1) and its potential interaction effects with household income on parental and child psychopathology, and 2) for children only, we assessed the included influence of both corresponding parental psychopathology subtypes and their potential interaction effects with household income on child psychopathology. Tukey’s tests were again applied for all significant effects. All tests were two-tailed with alpha levels set to p < .05, and significant results were Bonferroni corrected for multiple comparisons.

## Supplementary Material

1

## Figures and Tables

**F1:**
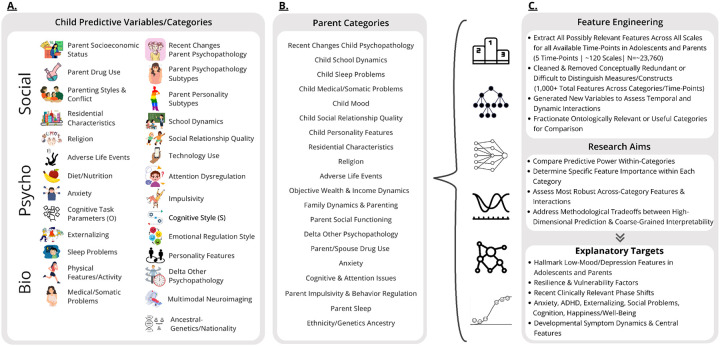
Methodological Overview: Categorical Feature-Sets Created for Predictive Models in Children and Parents, Brief Analytic Pipeline Overview (Figure 1. Ontological parcellation of feature-sets created for high-dimensional machine learning models, comprising 28 primary categories for children (A. left) and 20 for parents (B. middle), including over a thousand unique variables extracted and organized across 5 time points (see supplement S1.1 for individual measures for each feature-set and their associative structure in Fig 2 at time point 2). Categorical feature-sets were classified based on theoretical relevance or potential informative value for addressing uncertainty regarding hierarchical predictive capacities and differences for several hallmark psychiatric targets. Some categories included only child-self ratings (technology use), while many included parent and child ratings, and objective variable types when available. Some categories also included only objective variables which did not rely on any self-report. Another parcellation subsequently used for high-dimensional predictive comparisons included a spectrum of classically more objective variables (genetic PCs, neuroimaging, cognitive assessments, etc) towards subjective self or other ratings (see Fig F4b). Delta variables were also calculated for theoretically important measures when previous time-point data was available (ie. other psychopathology, social problems, family conflict, adverse life events, school problems, etc) to assess corresponding changes which predicted each other. Right panel (C.) briefly overviews the feature-engineering process, primary research aims, target outcomes evaluated, and methodological battery chosen to address tradeoffs between high-dimensional prediction with interpretability. Regarding high-dimensional prediction, our stacking machine learning ensemble included TABPFN, CATBoost, XGBoost, and Random Forests with a linear metamodel for dimensional targets and logistic regression metamodel for binary outcomes. Additional modelling included BMMs, CLPNs, and ANCOVAs, which utilized lower-dimensional theoretically driven variables for added interpretability, uncertainty estimation, and longitudinal analysis.)

**F2:**
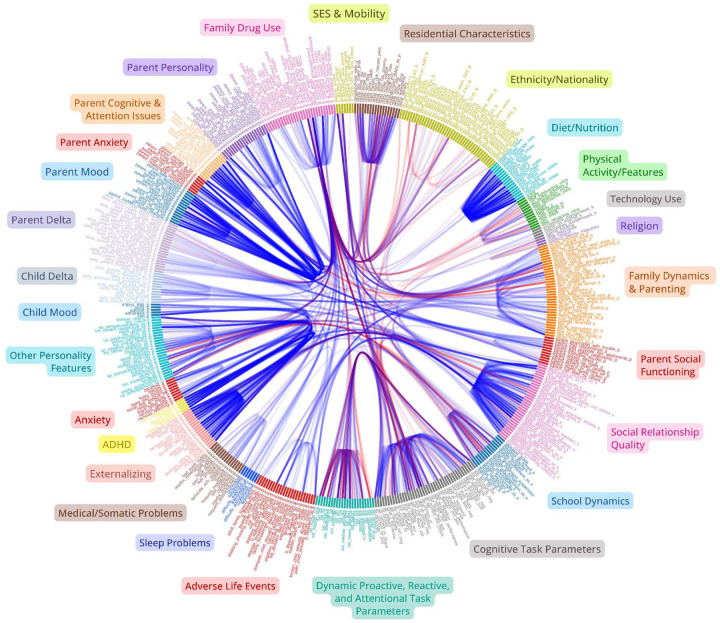
Associative Structure of Feature-Engineering Categories and Corresponding Measures used in Subsequent Predictive Models (Age 11 for Children) (Figure 2. Edge bundling diagram outlining relationships among all Biopsychosocial measures extracted *(excluding ~420 neuroimaging features)* in Children and Parents at one time point. Correlations outlined are from time point 2 (age 11 in children), represented here because it contained the widest range of collected variables. Each node represents a distinct predictor variable used in subsequent models, color-coded respectively by our categorization scheme. Edges indicate pairwise correlations, with blue representing positive correlations and red indicating inverse correlations. Edge opacity scales with correlation strength (alpha = abs(correlation) * 0.25), and bundling is applied (geom_conn_bundle, tension = 1.0) to improve interpretability by grouping related connections based on categorical domains. This visualization highlights the most interconnected features across the range of biopsychosocial measures (excluding neuroimaging) and categorical domains which were grouped for subsequent analysis at one of the time points. Several notable relationships are viewable within scales but have unexpectedly low correlations with some conceptually related constructs/categories.)

**F3:**
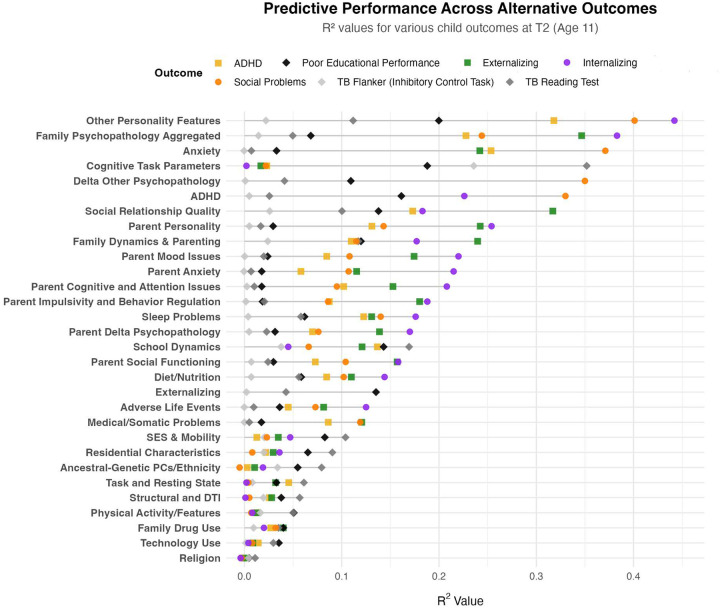
Within-Category Ensemble ML Regression Results for ADHD, Externalizing, Social Problems, Inhibitory Control Task, Reading Performance, and Educational Outcomes in Adolescents (Age 11) (Figure 3. Predictive performance (R^2^) across diverse biopsychosocial measurement categories for several child psychopathological and educational outcomes at age 11 (time point 2). All ML results used nested cross-validation (5 outer folds, 3 inner folds) with ensemble stacking of machine learning models (TABPFN, CATBoost, XGBoost, Random Forest, linear meta-learner). Outcomes assessed include ADHD, Externalizing, Internalizing, Social Problems, Poor Educational Performance (based on objective grade measures), NIH Toolbox (TB) Flanker inhibitory control task, and TB Reading Test performance. Categories demonstrating overly circular measures with outcome variables were excluded to maintain predictive validity (see methods). Notably, categories such as Changes in other Psychopathology Features (Delta), ADHD, and Personality Features consistently exhibited the strongest predictive relationships across psychopathological outcomes. Several categories, particularly more objective or biological measures (e.g., cognitive task parameters, neuroimaging, genetic ancestry principal components), showed predictive capacities for grade outcomes which were not notable in psychopathology targets. Conversely, all such objective or biological measures, despite showing moderate performance in grades and reading outcomes, were comparatively and noticeably minimal in predicting all psychopathology targets with some exceptions.)

**F3b:**
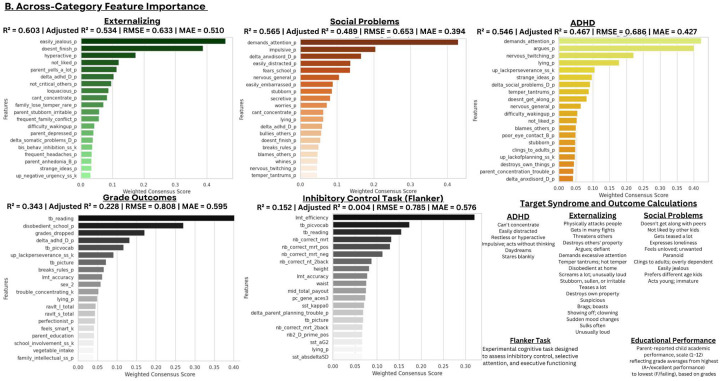
Across-Category Ensemble ML Performance and Feature-Importance Metrics (Age 11) (Figure 3b: Ensemble meta-model feature importance metrics from all features across categories after removing circular variables - revealing some distinct predictive patterns across diverse psychopathological, social, and cognitive outcomes. Externalizing showed the highest predictive accuracy in the ML ensemble (R^2^ = 0.603, Adjusted R^2^ = 0.534), with jealousy, failure to complete tasks, and hyperactivity as top predictors. Social problems (R^2^ = 0.565, Adjusted R^2^ = 0.489) and ADHD (R^2^ = 0.546, Adjusted R^2^ = 0.467) demonstrated similarly strong predictive performance, with attention-seeking appearing as a crucial predictor for both outcomes. Academic performance was used as a more objective target outcome to contrast and prediction proved more challenging (R^2^ = 0.343, Adjusted R^2^ = 0.228). The NIH Flanker task, a commonly used experimental measure of inhibitory control and attention, showed minimal predictive capacity (R^2^ = 0.132) despite using the same comprehensive feature set. The Adjusted R^2^ = 0.004 for the Flanker task indicates that the extensive number of predictors is introducing more noise than predictive signal - indicating likely validity concerns with the task and discrepancy between subjective-report and objective experimental tasks purported to measure similar constructs. These comparative results demonstrate that while psychopathological, experimental task, and behavioral outcomes share some common predictors (particularly attention-related features), they also show quite distinct predictive profiles across a range of biopsychosocial features. The consistently poor prediction of objective cognitive task performance across our analyses suggests fundamental limitations in the ecological validity of some experimental cognitive measures.)

**F4:**
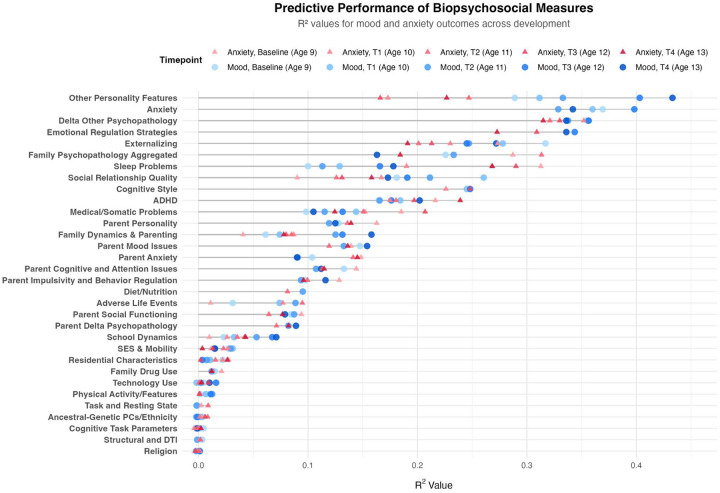
Hierarchical Within-Category Ensemble ML Regression Results for Low-Mood and Anxiety (across all Available Timepoints) in Adolescents (Ages 9–13): (Figure 4: Predictive performance (R^2^) of biopsychosocial measurement categories at all time available points across development (age 9 to 13) for target anxiety and hallmark low-mood/depression features (excluding secondary symptoms such as sleep and appetite). Results are again based on nested cross-validation (5 outer folds, 3 inner folds) with ensemble stacking machine learning models (TABPFN, CATBoost, XGBoost, Random Forest, linear meta-learner). Categories lacking sufficient data at specific time points were omitted. Strong predictive performance consistently emerged from categories such as Other Personality Features, Anxiety, Delta Other Psychopathology, Emotional Regulation Strategies, and Aggregated Family Psychopathology. Predictive strength notably increased across time with features extracted related to Social Relationship Quality. In contrast, objective biological measures, including Cognitive Task Parameters, Neuroimaging (Structural,DTI, Task, and Resting State), and Genetic Ancestry Principal Components, showed persistently limited predictive capacity across mood and anxiety outcomes. Due to reduced sample release as well as data pre-processing procedures, time point 4 (age 13 in children) was reduced to 3,327.)

**F4b:**
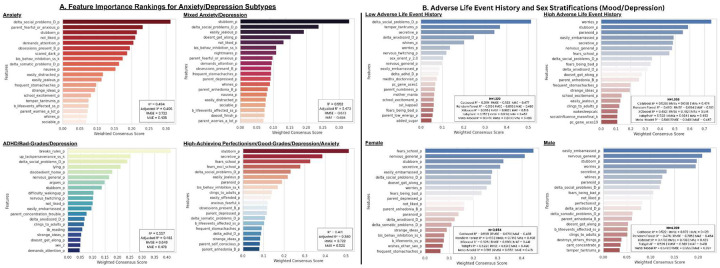
Across-Category Feature Importance Rankings for Various Mood/Depression/Anxiety Subtypes and Stratifications in Children (Age 11) (Figure 4b. Ensemble feature importance rankings across distinct mood/depression subtypes show both shared and unique predictor patterns. The mixed anxiety/depression model (R^2^ = 0.552, Adjusted R^2^ = 0.473) revealed stubbornness, recent changes in social problems, and jealousy as the strongest contributors, suggesting potential shared vulnerability factors across internalizing domains. Anxiety composite score model (R^2^ = 0.494, Adjusted R^2^ = 0.406) showed similar importance for social problems and stubbornness but had unique emphasis on parental anxiety

**F4c:**
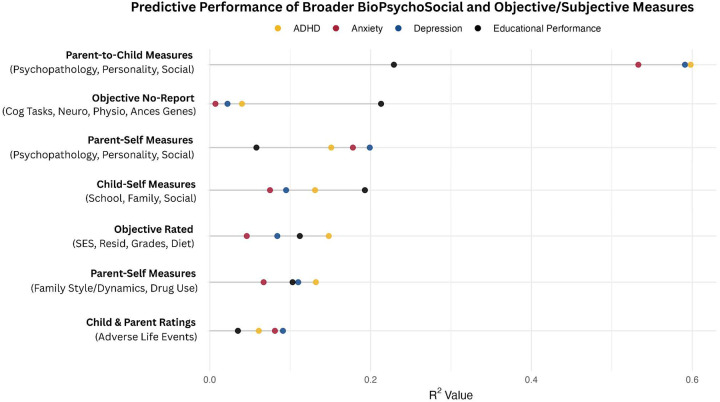
Further Biopsychosocial Objective/Subjective-Report Spectrum Fractionation: Ensemble ML Prediction of Depression, Anxiety, ADHD, and Educational Outcomes in Adolescents (Age 11) (Figure 4c: Predictive performance (R^2^) at age 11 (time point 2) for broader groupings of biopsychosocial also parcellated by objective versus subjective measures, again evaluated through nested cross-validation (5 outer folds, 3 inner folds) using ensemble stacking machine learning models. Variables were categorized based on their reliance on subjective or objective data: entirely objective (cognitive tasks, neuroimaging, genetic ancestry principal components, physiological/activity data), self-report but more objectively verifiable (SES, residential characteristics, grades, diet), child-self-report (school, family, social experiences), parent-to-child report (psychopathology, personality, social features), parent-self-report (psychopathology, personality, social, family dynamics, drug use), and jointly reported adverse life events.

**F5:**
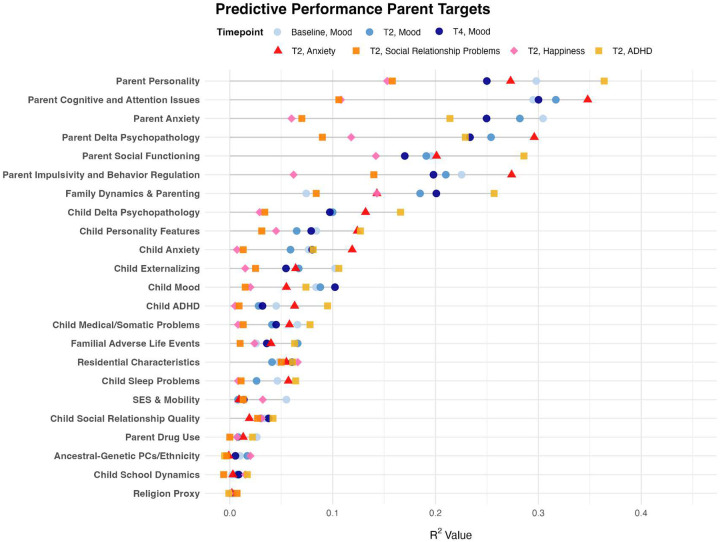
Hierarchical Within-Category Ensemble ML Regression Results for Low-Mood/Depression, Anxiety, Happiness, Social Relationship Problems, and ADHD in Adults/Parents (from all Available Timepoints): (Figure 5. Predictive performance (R^2^) of available biopsychosocial measurement categories for *parent* low-mood/depression (blue) outcomes across multiple time points (Baseline, T2, and T4). Secondary target features ADHD (yellow), social relationship problems (orange), anxiety (red), and happiness (pink) also included at T2 to provide additional operationalization of mood and contrasting psychopathological targets. Secondary mood symptoms (e.g., sleep, appetite disturbances) were again excluded from depression outcomes but were retained as predictors to focus specifically on core mood features. Categories with insufficient data at specific time points were omitted. Given the ABCD dataset’s focus on adolescent development data acquisition, several categories used for predictive models were not available or applicable for adults (e.g., cognitive tasks, neuroimaging, etc). Some measures variables used for children were used as proxies if applicable (e.g., religion, ancestral genetic PCs). Parents in this sample had a mean age of 40 and ~90% of data was collected from mothers.

**F6:**
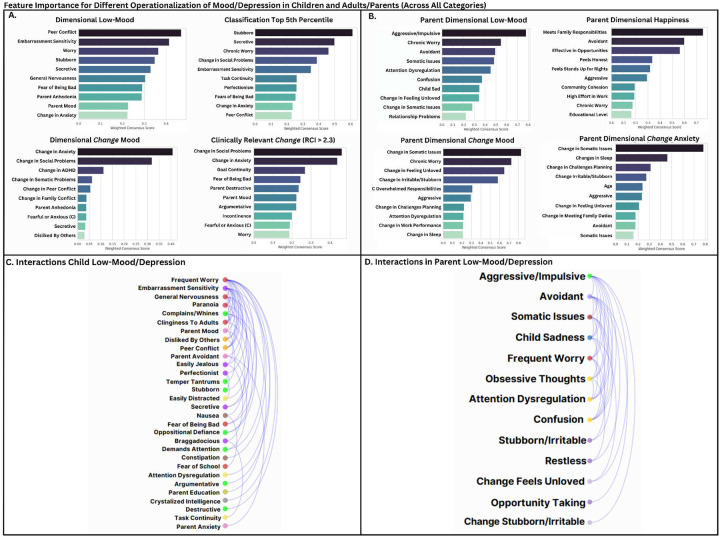
Across-Category Feature Importance Rankings for Different Operationalizations of Mood/Depression/Anxiety in Children and Adults [Figure 6. Ensemble features importance rankings across all categories for various operationalizations of mood in children (left) and parents (right) at time point 2. Rankings were derived from the meta-model in the ensemble stacking method, with features ordered by their contribution to predictive performance. Different operationalizations include dimensional symptom scores, clinically significant changes (RCI > 2.3), and severe cases (top 5th/10th percentiles). Color coding indicates feature hierarchy. SHAP values from all respective base-learner ML models are available in the supplement S3.1 and S3.2. Below: Most Robust interactions across all categories in adolescents (left) and parents (right) with SHAP values from best-performing ML models. Arc diagrams showing the strongest feature interactions predicting dimension of low mood/depression for children (left) and parents (right), based on SHAP interaction values from the best-performing ML model (CATBoost) at time point 2. Features are ordered hierarchically by nodal degree (number of significant interactions), displaying only interactions above the 95th percentile threshold. Highly interactive nodes for children include frequent worry, embarrassment sensitivity, general nervousness, and paranoia. In parents, and aggression, avoidant behaviors, somatic issues, and child sadness demonstrated strong interactions.]

**F7:**
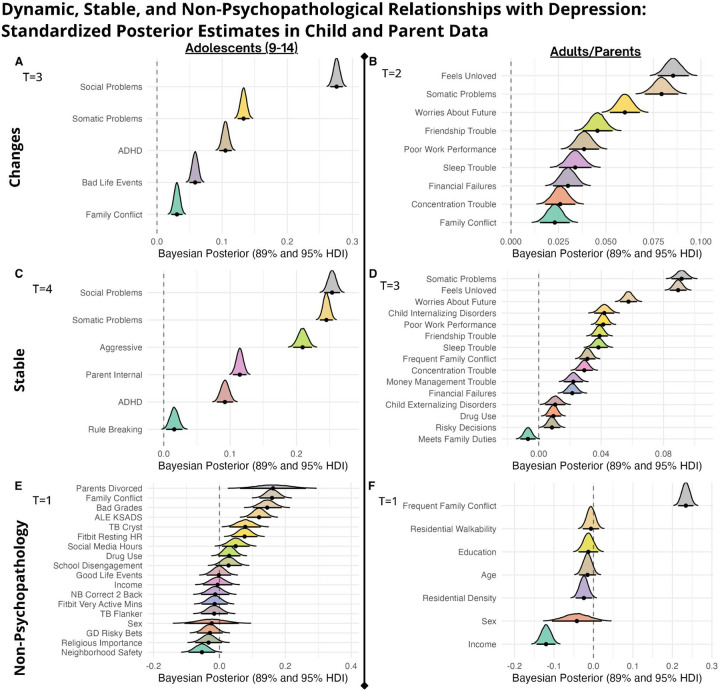
Bayesian Hierarchical Mixed Models of Dynamic, Stable, and Non-Psychopathological Relationships with Depression in Adolescents and Adults/Parents (Figure 7: Bayesian hierarchical mixed models showing standardized posterior distributions (89% and 95% HDIs) with CBCL depression outcomes in children (left panels A, C, E) and parents (right panels B, D, F) across available time points. Variables were selected based on theoretical relevance and availability over multiple assessments. Models separately estimate the strongest standardized effects for delta-change variables (top panels A-B), CBCL psychopathological measures (middle panels C-D), and broader non-personality/non-psychopathological measures (bottom panels E-F).

**F8:**
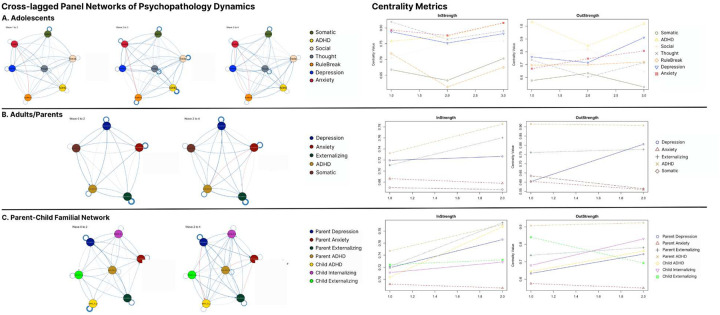
Cross-lagged Panel Network Models: Longitudinal Lower-Dimensional Psychopathological Interactions in Children, Parents, and Family (Figure 8. Autoregressive cross-lagged panel network models examining temporal relationships between CBCL psychopathological syndrome dimensions across available waves of data collection in children (Panel A, 4 waves), parents (Panel B, 3 waves), and parent-child interactions (Panel C, 3 waves). In both children and adults, ADHD consistently exhibited high OutStrength (right centrality metrics), signifying its influential role in the broader psychopathological network over time. For children, depression showed a pattern of increasing InStrength across waves, indicating heightened susceptibility to influences from other psychological domains as development progressed. Bidirectional positive relationships between depression and anxiety were consistent across all networks, confirming their closely intertwined development. Social problems in children maintained substantial connections with multiple symptom domains, particularly with depression. In the parent-child networks, parent ADHD and depression show notable influences on corresponding child symptoms. Child ADHD maintained high centrality even in the parent-child network, suggesting attentional dysregulation role in the potential nexus in familial psychopathology. The only consistent negative relationships observed were between anxiety and rule-breaking behaviors in children, potentially indicating divergent developmental pathways between internalizing and certain externalizing symptoms. These network patterns underscore the developmental significance of ADHD symptoms as a possible intervention target, given high Out and In-Strength centrality in child and parent networks. To ensure robustness of network findings, we implemented bootstrap procedures to assess stability of edges and centrality metrics. Edge weights were evaluated through 1000 bootstrap iterations, while centrality metrics were assessed using a case-dropping bootstrap procedure (2500 iterations) that systematically removes random subsets of the sample to quantify reliability under sampling variation. Comprehensive stability coefficients and detailed centrality metrics are provided in the supplementary materials S6.1.)

**F4Ex:**
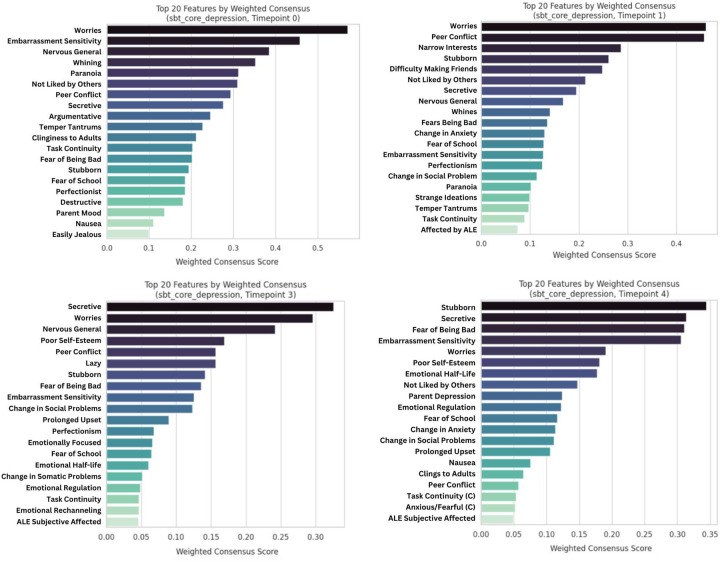
Ensemble ML Regression for All Additional Time Points: Top 20 Feature Importance Rankings for Child Low-Mood/Depression (Figure 4Ex: Top 20 features ranked by weighted consensus importance scores from ensemble machine learning models predicting child low-mood dimensional scores across four time points (T0, T1, T3, T4). Ensemble meta-model demonstrated consistent predictive performance across time points, with R^2^ values of 0.502, 0.545, 0.514, 0.584, and 0.605 for time points T0, T1, T2, T3, and T4, respectively. Features labeled “(C)” indicate child self-ratings rather than parent reports. Due to differences in available measures across assessment periods, additional BRIEF (Behavior Rating Inventory of Executive Function), emotional regulation, and social responsiveness scale features appear at specific time points (T1, T3, T4) which are not above. Several predictors consistently emerged across time, including secretive behavior, stubbornness, embarrassment sensitivity, worry, and negative social experiences, highlighting robustness as stability in adolescent low mood. Corresponding evaluation metrics and SHAP values for each model at every time point are included in the supplementary materials S3.2.)

**F7Ex:**
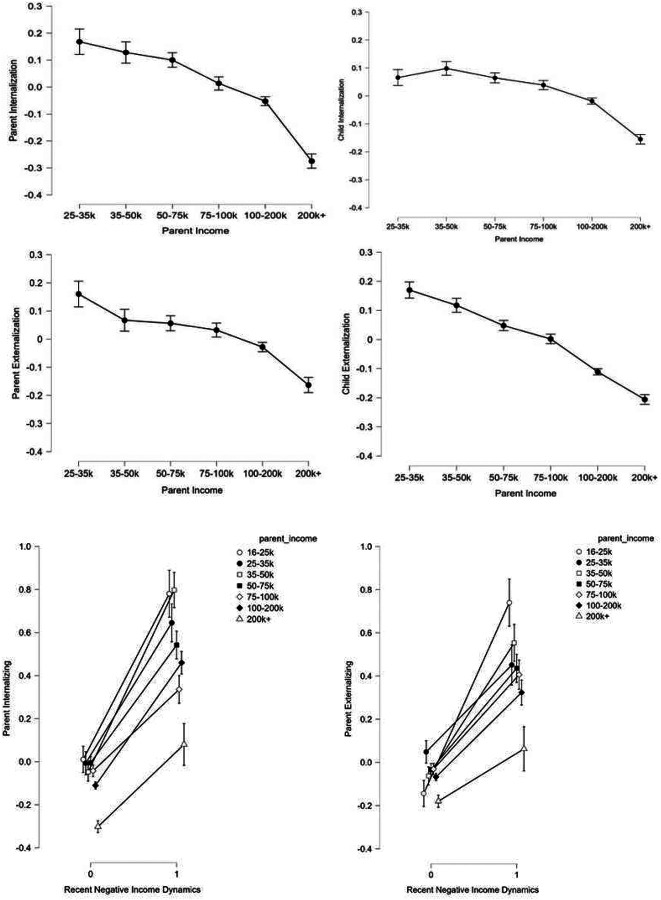
Differential Relationship of Family SES with Internalizing/Externalizing Outcomes in Parents versus Children and Objective versus Dynamic Income (Figure 7Ex: (A Top Figure.) Household income relationship with internalizing (top) and externalizing (bottom) psychopathology in parents (left) and adolescents (right). Objective household income was inversely associated with internalizing and externalizing psychopathology in both parents and their children. However, household income is more strongly associated with internalizing psychopathologies in parents and externalizing psychopathologies in children. (B Bottom Figure) Compared to objective family income (top), recent negative income dynamics exhibit even stronger relationships with parental internalizing and externalizing psychopathology (bottom). Y-axis represents adjusted T-scores. Error bars denote SEM.)

## Data Availability

Data used in the preparation of this article were obtained from the Adolescent Brain Cognitive DevelopmentSM (ABCD) Study (https://abcdstudy.org), held in the NIMH Data Archive (NDA). The data used in this study are available from the NIMH Data Archive. This is a multisite, longitudinal study designed to recruit more than 10,000 children age 9–10 and follow them over 10 years into early adulthood. The ABCD Study^®^ is supported by the National Institutes of Health and additional federal partners under award numbers U01DA041048, U01DA050989, U01DA051016, U01DA041022, U01DA051018, U01DA051037, U01DA050987, U01DA041174, U01DA041106, U01DA041117, U01DA041028, U01DA041134, U01DA050988, U01DA051039, U01DA041156, U01DA041025, U01DA041120, U01DA051038, U01DA041148, U01DA041093, U01DA041089, U24DA041123, U24DA041147. A full list of supporters is available at https://abcdstudy.org/federal-partners.html. A listing of participating sites and a complete listing of the study investigators can be found at https://abcdstudy.org/consortium_members/. ABCD consortium investigators designed and implemented the study and/or provided data but did not necessarily participate in the analysis or writing of this report. This manuscript reflects the views of the authors and may not reflect the opinions or views of the NIH or ABCD consortium investigators ABCD DOI: http://dx.doi.org/10.15154/abt7-zf22

## Code availability

All code used for preprocessing, modeling, and evaluation in this study, including ensemble machine learning scripts (e.g., Random Forest, Elastic Net, XGBoost, TabPFN), Bayesian mixed models (BMMs), cross-lagged panel networks (CLPNs), and Edge-Bundling, was written in Python or R. All code is available upon request and will be made accessible via a GitHub repository. Data access will be in accordance with ABCD data sharing agreements.
