# Supplementary material for: Hierarchical, Interactive, and Dynamic Predictive Capacity of Current Biological, Psychological, Social, and Environmental Measurements in Depression, Anxiety, ADHD, and Social Quality across the Lifespan
Source: Res Sq. 2025 Jul 30:rs.3.rs-7060126. Preprint. [Version 1] doi: 10.21203/rs.3.rs-7060126/v1 (PMC12324575; doi:10.21203/rs.3.rs-7060126/v1)
Supplement: 1 [file NIHPPRS7060126V1-supplement-1.pdf]

Supplementary Materials:

*.pdf or .doc formatting available upon request

Section S1: Full High-Dimensional Feature-Set Variables for Each Category, Time Point, Exact Scales Used, Individual Item Renaming

- **S1.1** Variables Extracted/Created, Scales, and Renaming
- **Extracted/Created ABCD Variables**

Section S2: Consensus Recommendations for Machine Learning in Science Reforms Checklist

- **S2.1** Methodological Standards Compliance (ML Reforms Checklist)
- **ML Reforms Checklist**

Section S3: ML for All Depression/Low-Mood Operationalizations, Neuroimaging, and Time Points: Full Within-Category Feature Importance, SHAP, Ensemble Stacking Evaluation Metrics, Individual Model Performance

- **S3.1** Low-Mood/Depression in Children and Parents: Time Point 2 Within-Category ML Results
- **ML T2 Within-Domain Child Depression Results**
- **S3.2** Low-Mood/Depression in Adoloscents: Across-Category ML and SHAP Results at All Time Points from All Models (Ages 9-13)
- **Ensemble ML Model Performance and SHAP Values - Low-Mood/Depression For Each Time Point**
- **S3.3** Parent-To-Child Reported Cognitive and Emotion Regulation Style Scales (Scale Names) (T1,T3)
- **ML Parent-To-Child Subjective-Report Cognitive/Emotion Scales**
- **S3.4** Parent Suicide Ideation: Across-Category ML Evaluation Metrics, Feature Importance, and SHAP Values(T0, T2, T4)
- **Across-Category ML Parent Suicide All Time Points**
- **S3.4** Neuroimaging Results for Different Mood/Depression Operationalizations and Social Vulnerability Index
- **ML Class Neuro Top 5 CBCL/KSADS Depression**

Section S4: Alternative Psychiatric and Social Targets: ML Within and Across Category Results

- **S4.1** Anxiety: Within-Category ML Results (T2)
- **ML T2 Within-Domain Child Anxiety Results**
- **(*** All further within-category analysis for all predictive targets is available upon request)
- **S4.2** ADHD, Externalizing, Social Problems, Mixed Anxiety/Low-Mood: Across-Category ML Results (T2)
- **Alternative Psychiatric Targets (ML Feature Importance and Model Performance)**

Section S5: Full ML Objective vs. Subjective Spectrum/Domain

- **S5.1** Objective-Self Report Spectrum Overview and Results, ML Metrics, SHAP Values
- **ML Objective - Self Report Spectrum T2**

Section S6: Bayesian Mixed Models, Cross-Lagged Panel Network Models, and ANCOVAs Extended Analysis

- **S6.1** Cross-Lagged Panel Network Results and Bootstrapping
- **CLPN Full Centrality and Bootstrapping Results**
- **S6.2** Full Bayesian Mixed Models Results across Mood/Depression Operationalizations (with contrasting ADHD outcomes using Non-psychopathological DVs)
- BMM Supp
- **S6.3** Extended Income-Psychopathology Relationships (ANCOVAs)
- **Income and Parent/Child Psychopathology Extra**
